# Next-Generation Sequencing Analysis Reveals Differential Expression Profiles of MiRNA-mRNA Target Pairs in KSHV-Infected Cells

**DOI:** 10.1371/journal.pone.0126439

**Published:** 2015-05-05

**Authors:** Coralie Viollet, David A. Davis, Martin Reczko, Joseph M. Ziegelbauer, Francesco Pezzella, Jiannis Ragoussis, Robert Yarchoan

**Affiliations:** 1 HIV and AIDS Malignancy Branch, Center for Cancer Research, National Cancer Institute, National Institutes of Health, Bethesda, Maryland, United States of America; 2 The Wellcome Trust Centre for Human Genetics, University of Oxford, Oxford, United Kingdom; 3 Institute of Molecular Oncology, Alexander Fleming Biomedical Sciences Research Center, Vari, Greece; 4 Nuffield Division of Clinical Laboratory Sciences, University of Oxford, Oxford, United Kingdom; 5 McGill University and Génome Québec Innovation Centre, Montréal, Canada; University of Southern California Keck School of Medicine, UNITED STATES

## Abstract

Kaposi’s sarcoma associated herpesvirus (KSHV) causes several tumors, including primary effusion lymphoma (PEL) and Kaposi’s sarcoma (KS). Cellular and viral microRNAs (miRNAs) have been shown to play important roles in regulating gene expression. A better knowledge of the miRNA-mediated pathways affected by KSHV infection is therefore important for understanding viral infection and tumor pathogenesis. In this study, we used deep sequencing to analyze miRNA and cellular mRNA expression in a cell line with latent KSHV infection (SLKK) as compared to the uninfected SLK line. This approach revealed 153 differentially expressed human miRNAs, eight of which were independently confirmed by qRT-PCR. KSHV infection led to the dysregulation of ~15% of the human miRNA pool and most of these cellular miRNAs were down-regulated, including nearly all members of the 14q32 miRNA cluster, a genomic locus linked to cancer and that is deleted in a number of PEL cell lines. Furthermore, we identified 48 miRNAs that were associated with a total of 1,117 predicted or experimentally validated target mRNAs; of these mRNAs, a majority (73%) were inversely correlated to expression changes of their respective miRNAs, suggesting miRNA-mediated silencing mechanisms were involved in a number of these alterations. Several dysregulated miRNA-mRNA pairs may facilitate KSHV infection or tumor formation, such as up-regulated miR-708-5p, associated with a decrease in pro-apoptotic caspase-2 and leukemia inhibitory factor LIF, or down-regulated miR-409-5p, associated with an increase in the p53-inhibitor MDM2. Transfection of miRNA mimics provided further evidence that changes in miRNAs are driving some observed mRNA changes. Using filtered datasets, we also identified several canonical pathways that were significantly enriched in differentially expressed miRNA-mRNA pairs, such as the epithelial-to-mesenchymal transition and the interleukin-8 signaling pathways. Overall, our data provide a more detailed understanding of KSHV latency and guide further studies of the biological significance of these changes.

## Introduction

Kaposi’s sarcoma-associated herpesvirus (KSHV; also known as human herpesvirus 8) is an oncogenic human γ-herpesvirus involved in the pathogenesis of several AIDS-associated malignancies, including Kaposi’s sarcoma (KS), primary effusion lymphoma (PEL) and multicentric Castleman’s disease (MCD) [1–4]. As with other chronic viruses, infection with KSHV results in a number of changes in the host cells. Many of these are mediated by KSHV to evade the innate and adaptive immune responses, prevent cell cycle arrest, inhibit apoptosis, modulate cellular signaling pathways, and facilitate lifelong infection in the host. Conversely, other changes occur as a result of the host defense response to viral infection.

MicroRNAs (miRNAs) are a subtype of small (~22 nucleotides) noncoding RNAs found in all metazoan eukaryotes [5]. Genes encoding miRNAs are primarily transcribed by RNA polymerase II, generating imperfect stem-loop hairpin structures that are then processed by the cellular proteins Drosha, DGCR8, and Dicer to produce a miRNA duplex. This duplex is then incorporated into an RNA-induced silencing complex (RISC) that binds one miRNA strand to messenger RNA (mRNA) targets, usually in the 3’ untranslated region (3’ UTR) and induces translational inhibition or degradation (for a review, see [6]). While the changes in gene expression due to a given miRNA are often modest, they are nevertheless important post-transcriptional regulators because a single miRNA has the potential to alter an entire biological pathway by inhibiting numerous mRNAs at once through partial base complementarity [6].

The human genome encodes thousands of miRNAs [7]. A limited number of viruses, including KSHV and other herpesviruses, also encode their own miRNAs [8]. KSHV expresses 12 viral precursor miRNAs located within the latency-associated region and these yield 25 mature miRNAs [9,10]. Being non-immunogenic, virally encoded miRNAs are potentially attractive tools for viruses as they can interfere with the host machinery without being detected by the host immune system.

In addition, a number of KSHV-encoded proteins can effect substantial changes in host cell gene expression, either by directly acting on one or more steps in protein expression, or by indirect mechanisms (e.g. cell signaling pathways). In particular, KSHV proteins expressed during viral latency, such as latency-associated nuclear antigen (LANA) or viral FLICE inhibitory protein (vFLICE), can directly induce changes in expression of certain mRNAs or miRNAs to facilitate the latent infection. The changes in cellular miRNA expression can in turn affect the expression of target genes. In addition, changes in cellular miRNAs may occur as part of the host response to viral infection.

Transcriptome profiling techniques such as RNA-sequencing and microarrays have been used to study PEL cells, which are a valuable model system for KSHV infection [11]. PEL lines are largely latent but can have some lytic KSHV gene expression. However, a limitation of this approach is the lack of otherwise identical uninfected control lines. To bypass this restriction, differential expression studies using microarrays or high-throughput sequencing techniques have explored other KSHV infection models, such as KS lesion samples and normal skin tissues [12–14], mock and KSHV-infected primary lymphatic endothelial cells [15,16], or the KS-derived epithelial SLK/SLKK (SLK+rKSHV.219) model [17,18]. Although previous studies have provided useful insights into KSHV infection and disease pathogenesis, to date there has been no report of a next-generation sequencing study combining both mRNA and miRNA expression profiles in KSHV-infected and uninfected control cells. Here, we present the outcome of a comprehensive analysis comprised of both small RNA-sequencing and poly-A enriched mRNA-sequencing in latently infected SLKK cells and uninfected control SLK cells. While these cells are derived from renal cancer and differ somewhat from the principal target cells in KSHV malignancies, advantages of this cell system are that SLKK cells can be compared to an uninfected counterpart and also that they have very low levels of spontaneous lytic KSHV replication. Thus we could focus on changes induced by latent infection, rather than those induced by an acute host response or expression of lytic KSHV genes. Furthermore, the combined analysis of miRNA and mRNA differential expression of SLK and SLKK cells enabled the identification of a number of viral-induced miRNA changes that were associated with expected inverse changes in the predicted target mRNAs, suggesting that miRNAs were mediating many of these changes. We further investigated the functions and pathways in which those miRNA-mRNA pairs were involved and highlighted their potential role in pathogen-influenced mechanisms. Overall, these findings provide evidence for a broad posttranscriptional regulatory network of miRNAs and their mRNA targets that will deepen our understanding of KSHV effects on the host cellular machinery and may inform further studies of specific miRNA-mRNA interactions in KSHV-infected cells.

## Material and Methods

### Cell culture

Human KS-derived SLK and SLKK cells (also known as SLK+rKSHV.219) [19–22] were a gift from Dr. Don Ganem (UCSF, CA). They were expanded on receipt, frozen in liquid nitrogen, and stored in a cryogenic tank until used in the experiments described hereafter. Cells were thawed and maintained in Dulbecco’s Modified Eagle medium supplemented with 10% v/v fetal bovine serum (Sigma-Aldrich, St Louis, MO) and 1% Penicillin/streptomycin/glutamine solution (Gibco, Carlsbad, CA). Additionally, KSHV-positive SLKK cells were periodically grown under selection with 10 μg/mL puromycin to maintain the viral episome. Not counting the time during which cells were frozen, SLK cells had been kept in culture for less than 3 months before being used for RNA isolation (RNA-Seq), transfection assays, or *de novo* KSHV infections. SLK cells infected with recombinant rKSHV.219 (SLKK cells) were originally selected over time using puromycin in order to reach a high latent viral expression. Therefore, again not counting the time that they were frozen, the SLKK cells were kept in culture for a longer period of time (6 months to a year) before being used for RNA isolation (RNA-Seq) and transfection assays. All experiments were done in triplicate independent cell cultures maintained at 37°C in humidified 5% CO_2_.

### *De novo* KSHV infection

The day prior to *de novo* KSHV infection, SLK cells were plated at 1x10^5^ cells per well in a 6 well-plate to obtain 50% confluence. Cells were incubated for 6 hrs at 37°C with DMEM containing 2% FBS, wild-type KSHV, and 8 μg/mL Polybrene (Hexadimetrhine bromide; Sigma-Aldrich). Cells were washed twice with PBS and maintained for another 5 days post infection (5 dpi) in normal growth medium before being harvested for RNA. When treated in this manner, 40% to 60% of the SLK cells have evidence of KSHV infection (by LANA staining) by day 5.

### Transfection of miRNA mimics

The day prior to transfection, SLK and SLKK cells were plated at 1x10^5^ cells per well in a 6 well-plate to obtain 50% confluence. The control scramble sequence (Negative control #1, Cat. No. 4464051) and the double-stranded miRNA mirVana mimics for miR-708-5p, miR-409-5p and miR-409-3p (assay ID MC11161, MC13028, and MC12446, respectively; Ambion, Life Technologies) were transfected at a final concentration of 10nM using DharmaFECT 1 (Dharmacon, GE Healthcare, Lafayette, CO) according to manufacturer’s protocol. Briefly, DharmaFECT 1 and Opti-MEM (1:50 v/v ratio) were incubated at room temperature for 10 min. Separately, Opti-MEM and 10 pmol of miRNA mimics or miR-scramble were incubated for 10 min at room temperature. 400 μL of these two mixtures (1:1 v/v ratio) were then incubated for 20 min at room temperature and subsequently added to the cells with 1.6mL of DMEM containing 10% FBS. Cells were harvested for RNA 48 hrs after transfection.

### RNA isolation

Total RNA was extracted from cells using miRVana miRNA isolation kit according to manufacturer’s instructions (Ambion, Life Technologies, Carlsbad, CA). RNA abundance and integrity were determined after isolation using a Nanodrop-ND-1000 spectrophotometer (Thermo Fisher Scientific, Waltham, Massachusetts, USA) and an Agilent 2100 Bioanalyzer (Agilent Technologies, Santa Clara, CA), respectively. Only samples of total RNA with an RNA integrity number (RIN) >9 were further used for RNA-Sequencing and small RNA-Sequencing. All samples were stored at -80°C.

### Preparation of microRNA libraries for deep sequencing

Small RNA libraries were constructed as previously described [23] using the TruSeq RNA Sample Prep kit (Illumina inc., San Diego, CA). Briefly, after running 2 μg of total RNA in a 15% urea-TBE gel (Invitrogen, Life technologies, Carlsbad, CA) for 1 hr at 200V, the 20 to 30 nucleotide RNA fraction was excised and eluted in 0.3 M NaCl. Following separation of the elute from the gel debris using a Spin-X-column (Thermo Fisher Scientific), the small RNA samples were precipitated in 100% ethanol and 1mg/mL glycogen, incubated at -80°C for 30 min, centrifuged at 14,000rpm for 25 min, washed with 75% ethanol, air dried and resuspended in RNAse-free water. Illumina TruSeq libraries were then prepared according to the manufacturer’s protocol and the final RNA library concentration was measured by a Qubit 2.0 fluorometer using Qubit dcDNA HS Assay kit (Life Technologies). We verified the size of the products contained in the libraries using a high sensitivity DNA chip and an Agilent 2100 Bioanalyzer (Agilent Technologies). Finally, a total of six miRNA libraries (three SLK and three SLKK samples) were sequenced using the Illumina HiSeq platform.

### Preparation of polyadenylated mRNA libraries for deep sequencing

Total RNA of samples used for small RNA-Sequencing was treated with Turbo DNA-free DNase I and Dynabeads (both from Ambion, Life Technology) to deplete samples from the residual DNA and to isolate the polyadenylated mRNA transcriptome, respectively. PolyA+ RNA libraries were then prepared with the ScriptSeq v2 RNA-Seq kit (Epicentre, Madison, WI). The final concentration and size distribution of the RNA libraries were measured by using a Nanodrop-ND-1000 spectrophotometer (Thermo Fisher Scientific), and by running a DNA 100 chip on an Agilent 2100 Bioanalyzer (Agilent Technologies). Finally, a total of six polyadenylated mRNA libraries (three SLK and three SLKK samples) were sequenced using the Illumina HiSeq platform.

### Bioinformatic analysis

For small RNA sequencing, reads were aligned against known miRNAs from miRbase (version 19.0) using the software package SHRIMP [24] (http://compbio.cs.toronto.edu/shrimp/README) with miRNA specific settings (-o 1-n 2-r 30%-h 50%—local-Q—qv-offset 32—sam). To process paired-end sequencing, reads were aligned separately covering the mature miRNA both on the forward and reverse read and the obtained number of matches were averaged. The match counts were normalized and tested for differential expression using the edgeR package [25] with default settings. For mRNA sequencing, adapter sequences were trimmed using the Fastx toolkit. Reads were aligned against two target genomes, human (hg19) and KSHV (Genbank accession number NC_009333.1) using TopHat [26] to generate spliced alignments. Transcripts were assembled using Cufflinks and Cuffdiff [27] in order to reveal differentially expressed genes. To be more conservative and eliminate infinite fold differences in cases where there was a nil denominator, fold changes were calculated adding 0.01 read to both the numerator and denominator. Significant mRNA fold change was determined by an adjusted P-value lower than 0.05 based on the Benjamini and Hochberg multiple testing correction. In order to visualize miRNA-Seq profiles, sequencing reads were converted to bedGraph format using BEDtools. The output files were then uploaded and displayed using the UCSC Genome Browser (http://www.genome.ucsc.edu). R (http://www.R-project.org) suite software was used for statistical analyses, heatmaps and scatter plots.

### Quantitative Real-Time PCR assays

For miRNA expression, stem-loop qPCR was performed using TaqMan Universal master mix (Applied Biosystems) and the following microRNA assays: 000512 for miR-210-3p, 001274 for miR-410-3p, 002099 for miR-224-5p, 002331 for miR-409-5p, 002332 for miR-409-3p, 002341 for miR-708-5p, 002342 for miR-708-3p and 461775 for miR-3614-5p. Stable under infection, reference RNA RNU43 (assay 001095) was used as endogenous control to normalize expression. Thermal cycling conditions included an enzyme activation step (95°C for 10 min) and 40 cycles of amplification at 95°C for 15s followed by 60°C for 1min.

For gene expression, cDNA synthesis was performed on total RNA using the Superscript II reverse transcriptase kit, 0.5mM dNTP set and 50 ng/μL random hexamer (Invitrogen). qPCR was performed using the FastStart Universal SYBR Green/ROX master mix (Roche Applied Science, Mannheim, Germany). Stable under infection, the 18S transcript was used as endogenous control to normalize expression. Thermal conditions included an enzyme activation step (95°C for 10min), 40 cycles of amplification at 95°C for 15 sec and 60°C for 1 min, and melting curve analysis following instrument standard instructions. The following primer sets were used: 5’-TTGCCGAAGATGAGACTGC-3’ and 5’-GCGTTCACCTTAACCAGCA-3’ for Caspase-2, 5’-AATTACTGTGGCCTACCAGGTGAATA-3’ and 5’-AAGCTGGCTAATTTTATCATTTCCA-3’ for Fibrinogen-beta, 5’-CCCTGGTCCCTACTCAACAA-3’ and 5’-CTGGACCCTGACACCCTAAA -3’ for LIF, 5’-GCAGTGAATCTACAGGGACGC-3’ and 5’- ATCCTGATCCAACCAATCACC -3’ for MDM2, 5’-GCGTCGAGCTGCTGAAGAGGC-3’ and 5’-ACTGGTGGTGGTGGAGGTGGA-3’ for Radixin, and 5’-GCCCGAAGCGTTTACTTTGA-3’ and 5’-TCCATTATTCCTAGCTGCGGTATC-3’ for 18S. All qPCR reactions were performed on an ABI 7300 Real Time PCR instrument (Applied Biosystems). Each experiment was performed in triplicate. Change in miRNA or mRNA expression was determined based on the delta Ct method [28].

### DNA isolation and PCR amplification

Genomic DNA was isolated from SLK and SLKK cells using the PureLink Genomic DNA Mini kit (Invitrogen) following the manufacturer’s instruction. DNA concentration and integrity were determined after isolation using a Nanodrop-ND-1000 spectrophotometer (Thermo Fisher Scientific, Waltham, MA). All samples were stored at -20°C. Eight individual regions of the 14q32 locus were probed using a set of custom-designed primers at 10μM (S1 Table) and AmpliTaq Gold 360 Master Mix (Applied Biosystems). DNA amplifications were performed in a final volume of 25μL containing 100ng of DNA template. The cycling conditions were as follows: 10min at 95°C, 35 cycles of amplification at 95°C for 30 sec, 60°C for 30 sec and 72°C for 1 min, followed by 7 min at 72°C. The final products were electrophoresed on a 1% agarose gel, stained with ethidium bromide and visualize under UV light.

### Pathway analysis and inverse correlation study of miRNA and mRNA expression levels

Pathway analysis and inverse correlations between expression levels of differentially expressed miRNAs and their respective target mRNAs were analyzed using Ingenuity Pathway Analysis (IPA, Ingenuity Systems, Redwood City, CA; www.ingenuity.com). This *in silico* analysis software reveals enrichment for molecular networks and signaling pathways. Moreover, we generated medium to high confidence miRNA target predictions and as well as experimentally observed miRNA-mRNA interactions using the IPA tool called “MicroRNA Target Filter” that integrates multiple target prediction algorithms such as TargetScan, TarBase, miRecords and the Ingenuity Knowledge Base. This allowed for the integration of miRNAs with mRNA targets, predicted and validated, that were both differentially expressed in SLKK vs. SLK cells. Opposite expression pairing between miRNA and mRNA levels was implemented to further refine the analysis. Further filtering options were applied such as the confidence parameter.

#### Gene set enrichment analysis (GSEA)

GSEA [29] was performed by the JAVA program using the c3 collection of Molecular Signature Database, MSigDB (http://www.broadinstitute.org/gsea/msigdb/index.jsp). 221 miRNA families are each linked to a separate gene set consisting of putative targets containing a complementary 3’-UTR miRNA binding motif. The input gene list was generated by our mRNA-Seq differential expression analysis and was tested for enrichment in targets belonging to individual miRNA target genesets. A P-value and a false discovery rate (FDR), estimating the statistical significance of enrichment, were calculated for each miRNA gene set.

## Results

### Distribution of cellular and viral miRNAs in KS-derived human SLKK cells by miRNA-Seq

**Fig 1:**
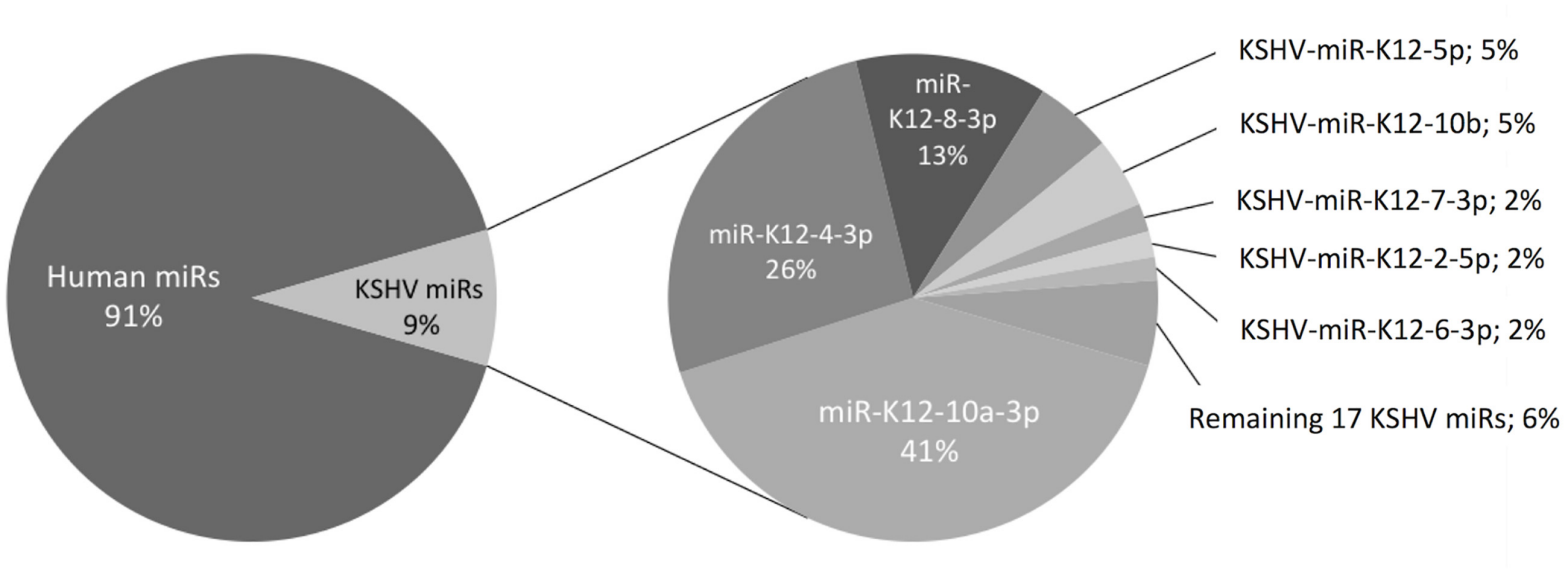
Relative abundance of human and KSHV-encoded miRNAs in KSHV-positive SLKK cells. The expression levels of KSHV miRNAs are shown as the percentage of the total of viral miRNA reads. Data are the mean of sequenced samples from three independent experiments, each with two technical replicates with opposite sequencing directions. Percentages have been rounded to the closest digit. For a detailed presentation, see S2 and S3 Tables.

Although KSHV only encodes for 25 mature miRNAs, they have been found to make up a significant proportion of the total mature miRNAs in PEL cells [11,30–32]. We analyzed cellular and KSHV viral miRNA expression in latently infected SLKK cells by deep sequencing. We observed that a sizable fraction (~9%) of miRNA reads in SLKK cells were of KSHV origin (S2 and S3 Tables). The distribution of the different KSHV miRNAs in SLKK cells (Fig 1) was generally similar to that of PEL cells BC-1, BC-3 and BCBL-1 reported previously [11]. For example, in SLKK cells, KSHV-miR-K12-4-3p and 8-3p comprised a similar proportion of KSHV miRNAs as in PEL cells (~26% and ~13%, respectively). However, there were some differences; KSHV-miR-K12-6-3p (2%) was expressed at a lower level, and KSHV-miR-K12-10a-3p (40.5%) was expressed at a higher level in SLKK cells compared with PEL cells (20–43% and <1%, respectively). It should be noted that substantial differences in KSHV miRNA distribution were previously observed even within the same cell type (B-cells, i.e. BC-1, BC-3 and BCBL-1) [11,30]. It is therefore not unexpected to observe similarities but also dissimilarities between the SLKK viral miRNA profile and that of PEL cells. Such differences may also arise from cell type differences between B-cells and SLK cells, the use of rKSHV.219 in the SLKK cells, or linker ligation biases.

### Comparison of expression profiles reveals more down-regulated than up-regulated cellular miRNAs in KSHV-infected cells

**Fig 2:**
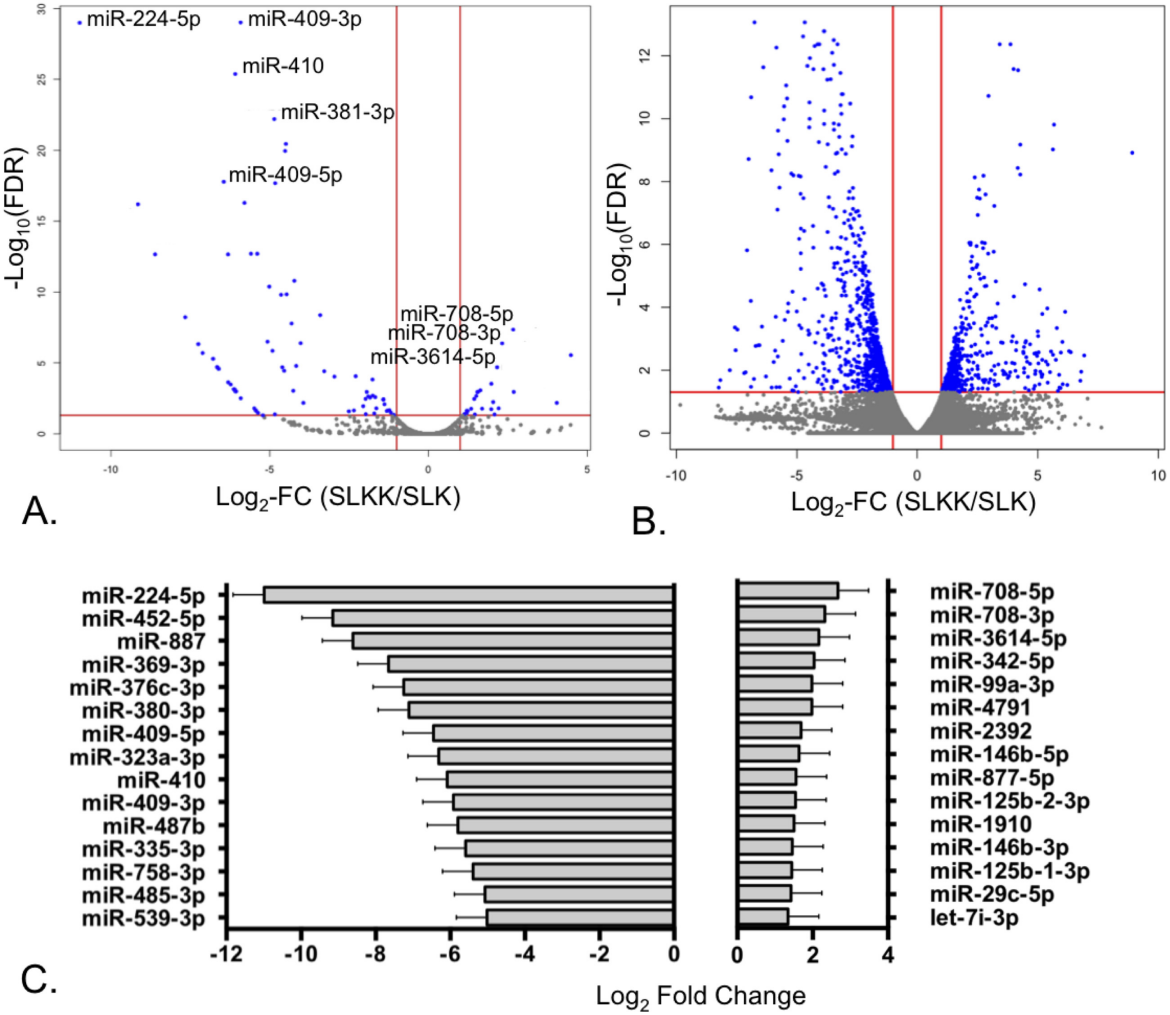
Expression of miRNAs and mRNAs in the analyzed libraries. A. Volcano plot of differentially expressed human mature miRNAs in KSHV-infected versus uninfected cells. Vertical red lines indicate the threshold for a relative expression fold change (FC) of 2 or -2 fold compared to uninfected controls. The horizontal red line represents the threshold of a 0.05 P-value. Thus, the blue points lying in the top right and top left sectors are significantly up-regulated and down-regulated, respectively, in KSHV-positive versus KSHV-negative cells (*P* <0.05, FC ≥2 or ≤-2). Selected miRNAs that have been validated by qRT-PCR are labeled. B. Volcano plot of differentially expressed mRNAs in KSHV-infected versus uninfected cells. The plot is depicted as in Fig 2A, with vertical and horizontal red lines similarly representing the thresholds of a fold-change of 2 or -2 fold, and of a false-discovery rate (FDR) of 0.05 respectively. C. Top 15 repressed and induced miRNAs in KSHV-positive vs. uninfected SLK cells with *P* <0.05 and medium to high miRNA expression (threshold of a mean miR count of 10 or more across all replicates).

We compared the cellular miRNA expression levels between the small RNA libraries of non-infected and KSHV-infected cell cultures. For the purpose of this analysis, we applied thresholds of ≥2 times fold change (FC) in expression (≤-1 and ≥1 log_2_ FC) and a P-value <0.05. Overall, we found 153 human mature miRNAs significantly altered by KSHV infection in SLKK vs. SLK cells (Fig 2A and S1 Fig). Interestingly, we observed substantially more suppressed than induced miRNAs, 111 vs. 42 respectively. In terms of expression fold change, this tendency was also apparent amongst the miRNAs with the greatest changes, which extended to an 11-fold down-regulation but only to a 4-fold up-regulation in log_2_ scale (Fig 2A). Amongst the most dysregulated miRNAs that were expressed in relatively high amounts (threshold of mean miR count > 10), miR-224-5p (-11 log_2_ FC), miR-452-5p and miR-887 (both -9 log_2_ FC), were significantly down-regulated, whereas the greatest relative induction was seen for miR-708-5p/3p and 3614-5p (all ~2 log_2_ FC) (Fig 2C).

### The 14q32 cluster is down-regulated by KSHV infection

It is noteworthy that 42 of the 111 miRNAs that were observed to be repressed by KSHV infection are located in the same genomic region, the 14q32 miRNA cluster (S2 Fig). For example, 14q32 miRNAs, miR-410, miR-409-5p and -3p, were significantly repressed -6 log_2_ FC upon KSHV infection (Fig 2C). Of note, while both arms of the miR-409 stem-loop were down-regulated, miR-409-3p had a higher relative abundance than miR-409-5p. Interestingly, there has been a previous report of frequent loss of 14q32 in PEL cell lines [33], which suggests that down-regulation of this region at the DNA level may be beneficial for the survival of KSHV-infected cells. To test whether the down-regulation in SLKK cells was similarly due to the loss of DNA encoding these miRNAs, we interrogated eight individual regions of the genomic DNA of SLK and SLKK cells by performing PCR (S3 Fig). We found that all tested portions of the 14q32 cluster were present in uninfected as well as KSHV-infected cells, suggesting post-transcriptional mechanisms lead to the substantial down-regulation of 14q32 miRNAs.

### mRNA-Seq analysis of SLKK cells

In parallel with these deep sequencing based miRNA studies, we also investigated the global changes in gene expression associated with chronic latent KSHV infection in order to assess the effects of the miRNA changes on their predicted targets. We compared the cellular mRNA expression levels of the poly-A enriched RNA libraries between non-infected SLK and infected SLKK cells. In all, 1,570 cellular genes, ~5% of all expressed genes, were significantly altered with either ≤-1 or ≥1 log_2_ FC (≥2 times linear FC) and an adjusted P-value (FDR) <0.05 (Fig 2B and S4 Fig). This included 980 repressed and 590 up-regulated mRNAs (Fig 2B). Some of these differentially expressed genes have been previously reported to be associated with KSHV infection. For example, in concordance with previous findings, DUSP1, JUN, and PLAUR were repressed whereas MDM2, CD55, CXCL1, IL6ST and ITGB4 were induced by KSHV infection [34–37]. Also, KSHV infection induced VCAM-1 (3 log_2_ FC), a vascular endothelial marker that has been linked to tumor angiogenesis and oncogenesis [38].

Some of the mRNAs that were decreased upon KSHV infection in SLKK cells have been previously described as targets for KSHV-encoded viral miRNAs. For example, the most abundant KSHV miRNA in SLKK cells, miR-K12-10a, is known to target TWEAKR (TNFRSF12A) [34], which was significantly repressed in SLKK vs. SLK cells (-2.0 log_2_FC and FDR = 0.001; Fig 2B). Another KSHV miRNA, miR-K12-11, down-regulates a translational regulator of interleukin 6, C/BEPβ [39], which was also decreased in SLKK compared to SLK cells (-1.3 log_2_FC and FDR = 0.016). It is quite plausible that the decreases in these target genes are the result of KSHV miRNA expression.

### Validation of miRNA differential expression patterns by Taqman assay

**Fig 3:**
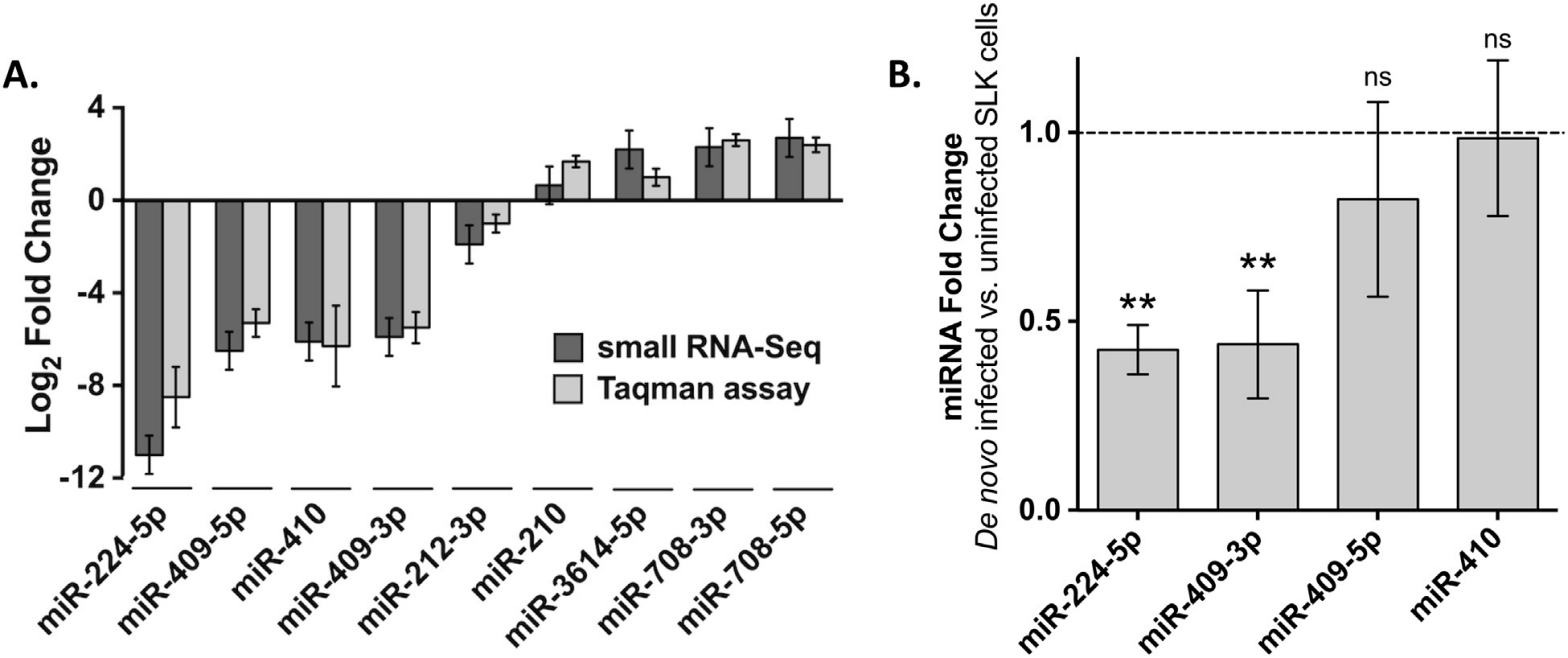
Analysis of miRNA expression in latent and *de novo* KSHV-infected cells. A. Data represent the average log_2_-transformed fold change of expression between latently infected SLKK cells and uninfected SLK cells, measured either by small RNA sequencing (black bars) or Taqman assay (grey bars). All the changes are significant (*P≤*0.05) except that of miR-210 as detected by miRNA-Seq. B. Effects of *de novo* KSHV infection on miRNAs seen down-regulated in the SLK/SLKK model. SLK cells were exposed to KSHV and after 5 days, changes in specific miRNAs were assessed by Taqman assay. The dotted line indicates the normalized level of these miRNAs in uninfected SLK cells. P-values were calculated using Student t-test. ** indicates P <0.01. ns: not significant. Expression levels of mature miRNAs were evaluated using the comparative CT method (2-deltaCT). Transcript levels of RNU43 were used for sample normalization. Bars depict the mean of 3 independent experiments; error bars reflect the standard deviation.

To validate the results observed in our miRNA sequencing data, we carried out Taqman assays (qRT-PCR) on several human miRNAs, with a focus on those with the greatest fold changes. Amongst the most significantly down-regulated miRNAs with high read counts were miR-224-5p, miR-410, miR-409-3p and -5p, whilst the most up-regulated miRNAs were miR-3614-5p, miR-708-3p and -5p. Because of the prominence of the miRNA repression, we also wanted to verify the differential expression of a miRNA that was less strikingly down-regulated, such as miR-212-3p. We also examined the change in miR-210 by qRT-PCR. This miRNA is of particular interest because it is induced by hypoxia-inducible factor (HIF) and because KSHV infection is known to increase HIF levels [40]. The levels of miR-210 by miRNA-Sequencing were slightly increased by KSHV infection, although the change did not achieve significance (P-value >0.05). Fig 3A shows the log_2_-transformed expression fold change of these miRNAs between SLKK and SLK cells, assessed by both miRNA-Sequencing and Taqman assay. The differential expression of all nine miRNAs tested by Taqman assay (eight of which met the RNA-seq P-value cut-off) was significant and consistent with our sequencing data. Pearson correlation coefficient (r) between the two measurement methods of the eight selected miRNAs indicates strong and significant positive correlation (r = 0.98, p < 10^–5^).

The relative expression of the 4 most down-regulated miRNAs seen in latently infected SLKK cells, 3 of which belong to the 14q32 cluster, was also examined following *de novo* KSHV infection of SLK cells. At 5 days post infection (5dpi), we found that 2 of these 4 miRNAs (miR-224-5p and miR-409-3p) were similarly down-regulated (n = 3) (Fig 3B). Also, miR-409-5p showed a trend for down-regulation, while miR-410 did not significantly change. A lower KSHV episome copy number in the *de novo* infected SLK cells as compared to SLKK cells could account for these changes. These results provide additional evidence that a proportion of the miRNA changes in SLKK vs. SLK cells are in response to viral infection. These results also suggest that some of the changes may only be observed in cells latently infected for a period of time.

### Investigation of miRNA target regulation

**Fig 4:**
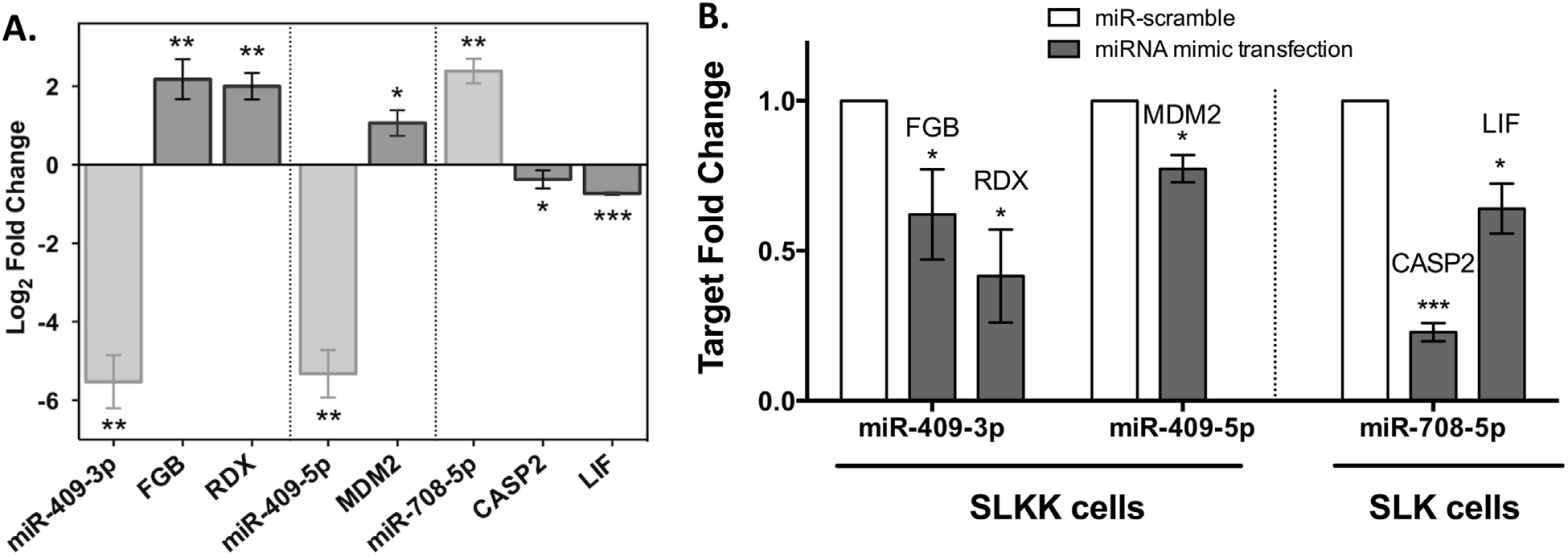
Differential expression of miRNAs and their known or predicted mRNA targets. A. miRNA expression in latently infected SLKK cells compared to uninfected SLK cells and the mRNA expression of their corresponding targets (Taqman and qRT-PCR assays). miRNAs and mRNAs are shown in light and dark grey, respectively. miR-409-3p targets fibrinogen beta (FGB) and radixin (RDX). miR-409-5p is predicted to target MDM2. miR-708-5p targets caspase-2 (CASP2) and is predicted to target leukemia inhibitor factor (LIF). P-values were calculated using Student t-test. *, ** and *** indicate P <0.05, 0.01 and 0.001, respectively. B. Effect of miRNA transfection on target regulation. Scramble control (miR-scramble) and miRNA mimics for miR-409-3p, miR-409-5p and miR-708-5p were transfected in either SLK or SLKK cells. Quantitative real-time polymerase chain reaction revealed the target expression for these miRNAs and the relative value is presented as the mean ± standard deviation based on 3 independent experiments. Statistical analysis and its representation are as in Fig 4A.

In an effort to correlate the expression levels of miRNAs to their respective target genes, we investigated known and predicted target mRNAs for three miRNAs validated by Taqman assay (miR-409-3p, miR-409-5p, and miR-708-5p). miR-409-3p, a miRNA down-regulated in our sequencing data, is known to target fibrinogen beta (FGB) and pro-metastasis gene radixin (RDX) [41,42]. The other arm, miR-409-5p, which is also down-regulated in SLKK vs. SLK cells, is predicted to target p53-inhibitor MDM2, according to Ingenuity Pathway Analysis (IPA) (Source: TargetScan Human). Our mRNA sequencing analysis indicated an induction of FGB, MDM2 and RDX (P-value = 8.6x10^-9^, 2.1x10^-3^, 1.6x10^-3^, respectively). This was also consistent with our qRT-PCR validation results showing a ~2 log_2_-fold induction for these three genes (Fig 4A).

Moreover, miR-708-5p, which was found up-regulated ~3 log_2_ FC by miRNA sequencing and ~2 log_2_ FC by qRT-PCR, is known to target pro-apoptotic protease caspase-2 (CASP2) [43]. Consistent with this, the qRT-PCR data demonstrated a small but significant reduction in CASP2 levels in KSHV-infected SLKK cells compared to control SLK cells. According to TargetScan, miR-708-5p is also predicted to bind the 3’UTR of leukemia inhibitory factor LIF mRNA and indeed we found it to be significantly down-regulated by qRT-PCR (Fig 4A). The strong reproducibility between both our miRNA-Seq and mRNA-Seq, and our qRT-PCR analyses provides evidence that the RNA-Seq was very robustly analyzed and enabled a valid assessment of the miRNA and gene expression profiles.

In order to further investigate whether the changes in mRNA targets in SLKK cells could be a result of differential miRNA expression, we carried out transfection assays to re-express miRNAs that were decreased in either SLKK cells (miR-409-3p and -5p) or SLK cells (mir-708-5p), as compared to their counterparts. Transfection of miR-409-3p in SLKK cells led to a decrease in two of its known targets, fibrinogen beta and radixin. Additionally, transfection of miR-409-5p in SLKK cells led to a significant decrease in the expression of its predicted target, the p53-inhibitor MDM2. This is the first report providing experimental evidence that miR-409-5p targets MDM2. Finally, transfection of miR-708-5p in SLK cells resulted in a dramatic (80%) decrease in its target, caspase-2; it also led to the significant decrease of predicted target LIF (~40%). This provides evidence that the up-regulation of miR-708-5p in SLKK cells is likely causing the observed down-regulation of caspase-2 (Fig 4B).

### Two integrated analyses of miRNA and mRNA show post-transcriptional regulatory networks relevant to KSHV pathogenesis

Based on the above results, we hypothesized that at least some of the changes in mRNA expression levels in infected cells are caused by the differentially expressed miRNAs. We further hypothesized that these miRNA-mRNA pairs may play a role in KSHV infection or in the cellular response to chronic virus infection, and also in the pathogenesis of KSHV-related diseases. To explore this, we used MSigDB, a function of Gene Set Enrichment Analysis (GSEA), to investigate the enrichment of the differentially expressed genes in targets belonging to distinct miRNA families. A miRNA family is determined by its seed region, a 6–8 nucleotide sequence that is located at the 5’ end of the miRNA and is able to bind target mRNA, promoting its degradation. Using our RNA-Seq data, we identified both up-regulated and down-regulated genes in the SLK/SLKK model. We then compared the GSEA output miRNA list to our miRNA-Seq data and selected for those miRNAs whose up-regulation is associated with the observed target down-regulation and then those whose down-regulation was associated with target up-regulation. This analysis allowed us to pair 4 up-regulated miRNAs with 74 down-regulated targets and 17 down-regulated miRNAs with 195 up-regulated targets overall (Table 1). These findings support the hypothesis that some of the changes in gene expression from cells latently infected with KSHV are caused by reciprocal expression changes in the miRNA that target those genes.

**Table 1:** miRNAs predicted to target differentially expressed mRNAs using GSEA.

miRNA	Target Sequence	Putative target genes (T)	Genes in Overlap (O)	Ratio O/T	P-value	FDR
**Up-regulated miRNAs/Down-regulated targets**						
miR-29c-3p	TGGTGCT	521	33	0.0663	1.40x10^-8^	3.09x10^-7^
let-7i-5p	CTACCTC	391	25	0.0639	6.53x10^-7^	6.87x10^-6^
miR-326	CCCAGAG	155	13	0.0839	1.94x10^-5^	1.07x10^-4^
miR-503-5p	CGCTGCT	24	3	0.125	1.24x10^-2^	2.75x10^-2^
**Down-regulated miRNAs/Up-regulated targets**						
miR-203a-3p	CATTTCA	287	29	0.1010	4.07x10^-19^	8.99x10^-17^
miR-148a-3p	TGCACTG	304	22	0.0724	7.03x10^-12^	1.41x10^-10^
miR-212-3p	GACTGTT	161	13	0.0807	3.91x10^-8^	2.88x10^-7^
miR-369-3p	GTATTAT	207	14	0.0676	1.09x10^-7^	7.55x10^-7^
miR-128-3p	CACTGTG	337	17	0.0504	3.38x10^-7^	2.02x10^-6^
miR-377-3p	TGTGTGA	200	13	0.0650	4.88x10^-7^	2.70x10^-6^
miR-452-5p	GAGACTG	94	9	0.0957	1.17x10^-6^	5.63x10^-6^
miR-323a-3p	TAATGTG	160	11	0.0688	2.14x10^-6^	9.85x10^-6^
miR-380-3p	ATTACAT	102	9	0.0882	2.33x10^-6^	1.05x10^-5^
miR-381-3p	CTTGTAT	206	11	0.0534	2.36x10^-5^	8.83x10^-5^
miR-7-5p	GTCTTCC	169	9	0.0533	1.31x10^-4^	4.21x10^-4^
miR-485-3p	TGTATGA	153	8	0.0523	3.49x10^-4^	9.88x10^-4^
miR-224-5p	GTGACTT	158	8	0.0506	4.32x10^-4^	1.19x10^-3^
miR-409-3p	AACATTC	142	7	0.0493	1.14x10^-3^	2.90x10^-3^
miR-205-5p	ATGAAGG	157	7	0.0446	2.03x10^-3^	4.73x10^-3^
miR-134-5p	CAGTCAC	52	4	0.0769	2.74x10^-3^	6.18x10^-3^
miR-299-3p	CCCACAT	54	4	0.0741	3.14x10^-3^	6.94x10^-3^

Using our list of differentially expressed mRNAs, we predicted which miRNAs would be upstream inhibitors with the Molecular Signatures Pathways Database (MSigDB). The overlap between the MSigDB output and our miRNA-Seq data highlighted 21 miRNAs that were differentially expressed in SLKK compared to SLK cells. Columns identify their names, the target sequence used to identify base complementarity with the input mRNA, the total number of putative target genes for a given miRNA, the number of putative targets that overlap with our input mRNA list, the percentage ratio between the overlap and the total number of putative targets, and its associated P-value and false discovery rate (FDR), correcting for multiple hypothesis testing.

**Fig 5:**
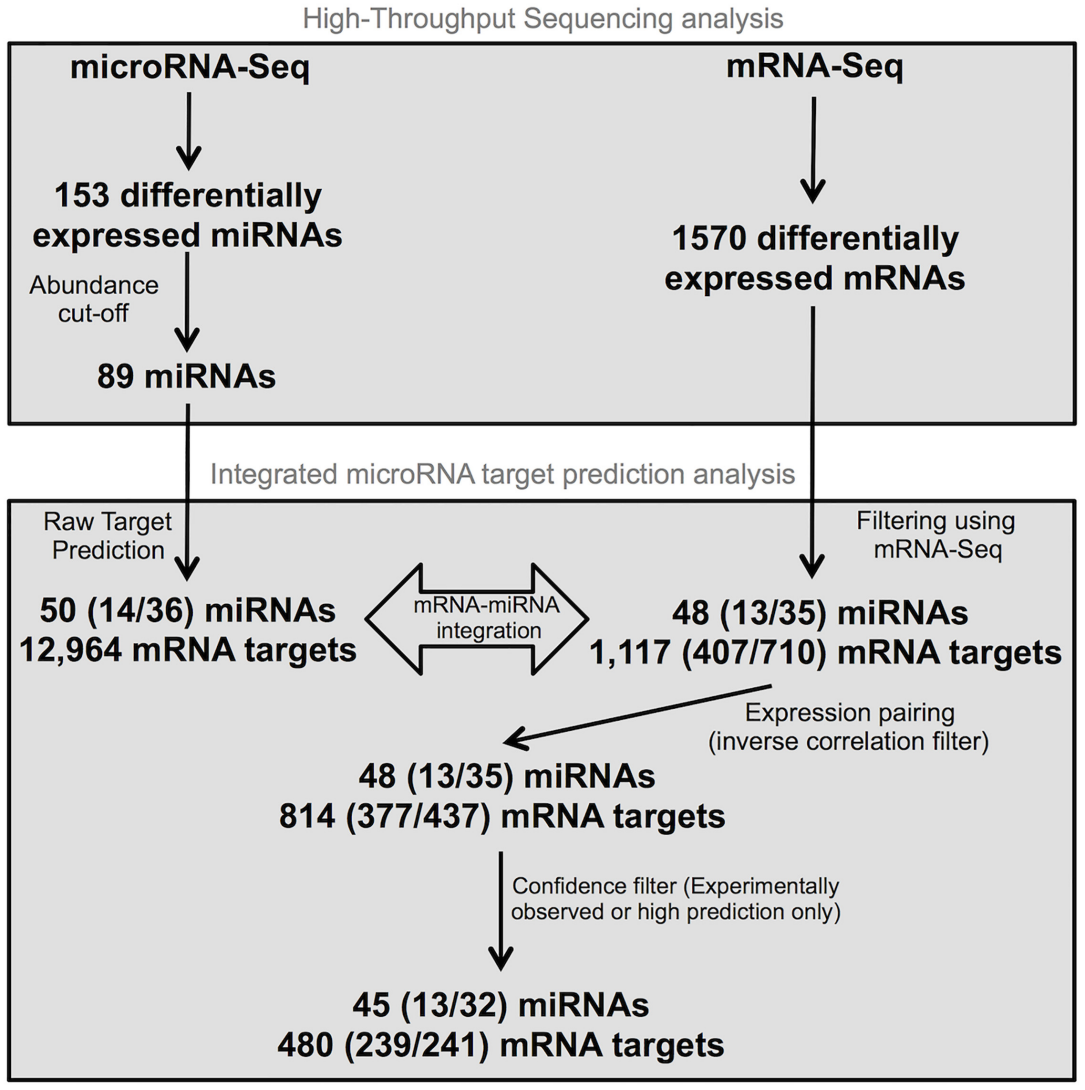
Workflow of integrated miRNA-mRNA association analysis using IPA. This experimental workflow shows the various filters used to associate miRNA-Seq and mRNA-Seq data. Numbers are presented as Total differentially expressed miRNAs or mRNAs (Up-regulated/Down-regulated).

A complementary approach for integration of miRNA and mRNA datasets is to use Ingenuity Pathway Analysis (IPA), which has an extensive collection of both known and predicted targets. IPA gathers comprehensive miRNA targeting information by combining multiple target prediction tools (TargetScan, miRecord and TarBase). Fig 5 summarizes the IPA data mining process using both sequencing datasets. Because miRNAs are known to effect relatively small changes in target mRNA expression and because changes induced by poorly expressed miRNAs might be particularly hard to detect at the target level, we focused on the medium to highly expressed miRNAs that were significantly affected by KSHV infection.

Of the 153 differentially expressed miRNAs, 89 met the read count cut-off of 60 reads across all replicates (i.e. an average of 10 per replicate). IPA then identified 50 of these 89 miRNAs that had either predicted or validated targets (a total of 12,964 targets). Of these 50 miRNAs, 48 had targets in the list of differentially expressed (≥2 times linear FC and FDR <0.05) mRNAs generated by our sequencing analysis, for a total of 1,117 targets. Interestingly, each of the 48 miRNAs targeted at least one gene that was affected in the expected direction. In total, the differential expression of 73% of those target genes (814 out of 1,117) was inversely correlated to that of their respective miRNAs, suggesting that at least a portion of the differential mRNA expression was a result of changes in miRNA expression.

**Fig 6:**
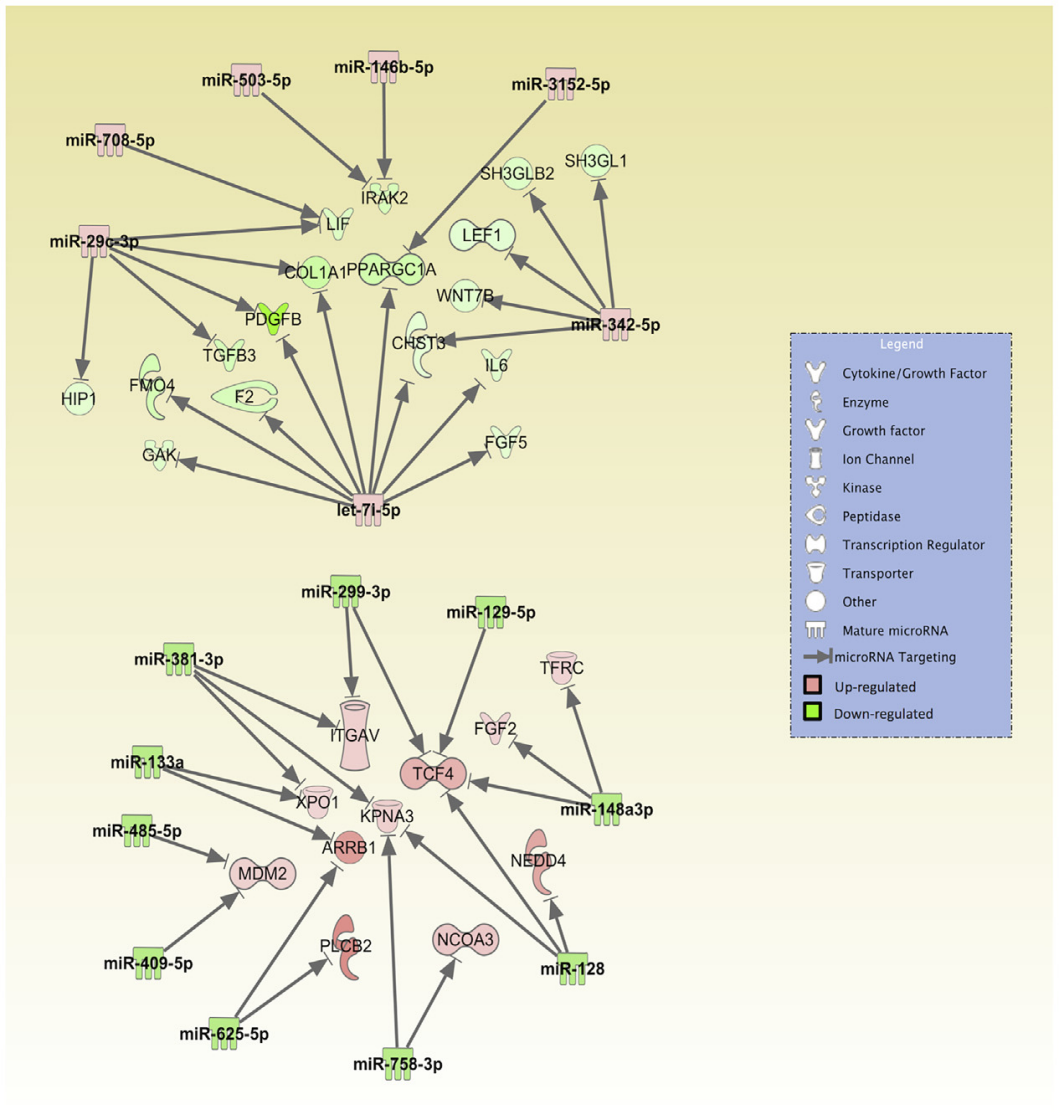
Implications of miRNA-mRNA pairs in Pathogen-Influenced Signaling. A total of 17 miRNAs and 28 mRNA targets are involved in the super pathway entitled ‘Pathogen-Influenced Signaling’. Only experimentally validated and highly predicted targets are presented here. The top part shows the up-regulated miRNAs and respective down-regulated targets. The bottom part shows the down-regulated miRNAs and respective up-regulated targets. The intensity of colors represents the extent of the up-regulation or down-regulation, in red and green respectively.

To adopt a conservative approach, we further filtered our dataset using experimentally observed or highly predicted target correlations. After this filter, we still had 45 miRNAs targeting 480 mRNAs, with each paired interaction being inversely correlated (Table 2 and Fig 5). Of these, 8 miRNAs were paired with 34 target genes that had been experimentally determined (Table 3), and 17 miRNAs had targets directly involved in pathogen-influenced signaling pathways (Fig 6). We calculated that a third of IPA miRNA filter results overlapped with those of MSigDB. Also, two third of the MSigDB matched IPA analysis, confirming the effectiveness of the IPA analysis.

**Table 2:** Ingenuity analysis predicts inversely correlated miRNAs and mRNAs pairs.

miRNA	Log_2_ Fold Change	# Target gene	Name of top 5 target genes
**Up-regulated miRNAs/Down-regulated targets**			
miR-708-5p	2.67	17	FOXN3, RGL1, POU6F1, RNF150, PAPPA
miR-3614-5p	2.16	2	FOXN3, MERTK
miR-342-5p	2.04	37	VANGL2, NR4A1, KIF21B, GNG4, ITPRIP
miR-146b-5p	1.64	19	NRIP3, SDK1, ZNF90, SYT1, KCNIP3
miR-877-5p	1.56	2	SUSD5, PNPO
miR-146b-3p	1.46	12	VANGL2, TSKU, CYFIP2, ASB6, ZNF488
**miR-29c-3p**	1.23	81	NAV2, C1orf21, FOXN3, GPR37, DUSP2
miR-3664-3p	1.21	7	SPOCK1, PNMA2, CD6, CBX6, FAM109A
miR-3152-5p	1.18	6	SRSF8, CTNNA2, CSPG5, PPARGC1A, SLITRK4
**miR-503-5p**	1.17	20	C1orf21, KIF5C, MOB3B, APLN, MYOM3
miR-4745-5p	1.10	13	FBXL7, LONRF2, ST6GALNAC3, MOB3B, GAREM
**let-7i-5p**	1.04	86	C1orf21, FOXN3, TRHDE, LIN28B, PDGFB
miR-21-3p	1.03	4	LIN28B, SHC3, ANO5, FAM117A
**Down-regulated miRNAs/Up-regulated targets**			
**miR-224-5p**	-10.99	23	SLCO2B1, FGB, CAST, RNF144B, DPP8
**miR-452-5p**	-9.15	4	FAM8A1, STAM2, MTMR6, PNN
miR-376c-3p	-7.25	6	TGFBR3, FHDC1, SLC7A11, SECISBP2L, TBC1D12
**miR-380-3p**	-7.11	1	LPP
miR-409-5p	-6.45	4	FAM102B, TBC1D15, MDM2, SRSF11
**miR-323a-3p**	-6.31	1	KIAA1430
miR-758-3p	-5.39	14	C2CD4A, NTM, RAB27B, NUDT12, NCOA3
**miR-485-3p**	-5.07	3	GOLGA8A/GOLGA8B, UBA6, ZMYM4
**miR-381-3p**	-4.86	53	ID4, SLCO2B1, PTGFR, UNC5C, ICK
miR-411-5p	-4.52	2	NR3C1, RYBP
miR-654-3p	-4.49	2	GMFB, ZNF91
miR-323b-3p	-4.47	3	SPAST, EIF1AX, SMIM13
miR-133a	-4.23	33	HAPLN1, ID4, NDRG1, PLXNA2, ARRB1
miR-654-5p	-4.03	8	LZTS1, UNC5C, FAM8A1, PDGFRB, STK38
miR-369-5p	-4.17	1	ID4
miR-485-5p	-3.28	13	NTM, MLLT3,ZMF527, PLEKHA5, UPF2
**miR-299-3p**	-2.96	4	TCF4, SLC16A7, SP4, ITGAV
**miR-205-5p**	-2.29	27	GATA3, GSE1, AXIN2, UNC5C, SECISBP2L
miR-3173-5p	-2.05	4	ADAMTS15, STC2, PEAK1, SKP1
miR-1914-5p	-1.97	3	SKIDA1, GLS, PAQR8
**miR-212-3p**	-1.89	21	HAPLN1, BTG2, ENPP4, CTDSPL2, KDM7A
miR-129-5p	-1.84	21	NTM, GSE1, ARRDC3, TCF4, CTDSPL2
miR-2355-5p	-1.64	10	CDH1, PLXNA2, LIMA1, ELF3, BTG2
**miR-148a-3p**	-1.61	45	SPTLC3, ADAMTS15, SKIDA1, ARRDC3, SLC7A11
miR-4786-5p	-1.49	5	PLEKHA5, SNRNP48, TRIM59, COG6, SSR1
miR-149-5p	-1.42	14	ID4, CD34, SORT1, MCTP2, PAQR5
miR-625-5p	-1.31	13	RUNX3, PLCB2, SPN, ARRB1, AXIN2
miR-450a-5p	-1.23	1	DUSP10
miR-548h-5p	-1.15	2	EDIL3, SMAD5
miR-4677-3p	-1.13	1	RPS27L
miR-196b-5p	-1.09	14	TGFBR3, DCDC2, FAM102B, PTPRG, BLOC1S6
**miR-128-3p**	-1.02	49	HAPLN1, TGFBR3, SLC35F3, DTX4, NEDD4

Using our lists of differentially expressed miRNAs and mRNAs as input for the Ingenuity Pathway Analysis (IPA), we found that 45 differentially expressed miRNAs target 480 mRNAs (medium and high confidence). Only mRNAs and miRNAs with opposite differential expression are shown in this table. Columns identify the miRNA name, its log_2_-transformed expression fold change between SLKK and SLK cells, the number of identified targets, and the five or less most differentially expressed targets. In bold are miRNA prediction overlapping with GSEA analysis (Table 1).

**Table 3:** miRNA-mRNA pairs found inversely correlated and validated in the literature.

miRNA	Known Target	Reference
**Up-regulated miRNAs/Down-regulated targets**		
let-7i-5p	ATAD3B, BCL2L1, CCND1, CDIPT, DUSP23, FAM105A, F2, GAK, GPR56, GYS1, MARS2, POLD2, POM121/POM121C, RHOB, RHOG, TAGLN	Selbach et al., 2009 (Nature)
miR-29c-3p	COL1A1, DUSP2, GPR37, KLF4, LOXL2, MAPRE2, MYBL2, TGFB3	Li et al., 2009 (J Biol Chem)
miR-146b-5p	IRAK2, NOVA1, PTGES2, SYT1	Hou et al. 2009 (J Immunol)
miR-503-5p	CCND1	Jiang et al., 2009 (BMC Cancer)
miR-485-5p	MDM2	Ratoviski et al., 2014 (Cell Cycle)
miR-708-5p	CASP2	Song et al., 2013 (J Cancer Res Clin Oncol)
**Down-regulated miRNAs/Up-regulated targets**		
miR-129-5p	TNPO1	Dyrskjot et al., 2009 (Cancer Res)
miR-205-5p	ZEB1	Gregory et al., 2008 (Nat Cell Biology)
miR-409-3p	FGB, RDX	Fort et al. 2010 (Blood)

This table shows the association between miRNAs and mRNAs that were significantly dysregulated in SLKK cells compared to SLK cells. Only the miRNA-mRNA pairs experimentally validated in the literature are shown here. The majority of the targets were identified through IPA-integrated TarBase v5.0 and others independently.

Using these 480 differentially expressed mRNAs as input, pathway analysis revealed an enrichment in several signaling pathways that could be relevant to KSHV pathogenesis, such as “regulation of the epithelial-to-mesenchymal transition pathway”, “IL-8 signaling”, “clathrin-mediated endocytosis signaling” and “Wnt/β-catenin signaling” (Table 4). Of note, EMT plays a central role in cancer progression and can be promoted by KSHV through Notch-induced ZEB1, a transcription factor up-regulated in our dataset [45]. Also, the Wnt/-catenin pathway, which regulates tumor development and cell proliferation, is altered by latency-associated nuclear antigen LANA, a major KSHV latency protein suggesting multiple viral mechanisms that achieve the same result [46]. Finally, the enrichment of the interleukin-8 signaling pathway is noteworthy in that KSHV ORF74, which plays a key role in KS pathogenesis, is a constitutively active viral homologue to the interleukin-8 receptor. Altogether, these findings further support the role of miRNAs and their transcript targets in KSHV pathogenesis and/or the response to cells to chronic viral infection.

**Table 4:** Canonical pathways affected by differentially expressed miRNA-mRNA pairs.

Ingenuity Canonical Pathways	P-value	Ratio (% of genes in pathway)	Dysregulated target gene	Dysregulated miRNA
Regulation of theEpithelial-to-Mesenchymal Transition Pathway	2.5 x 10^–3^	12/182 (6.6%)	**CDH1**, **FGF2**, FGF5, FGFRL1, **ID2**, LEF1, **PARD6B**, **PDGFRB**, **TCF4**, TGFB3, WNT7B, **ZEB1**	**let-7i-5p**, **miR-29c-3p**, miR-128, miR-129-5p, miR-148a-3p, miR-196b-5p, miR-205-5p, miR-299-3p, **miR-342-5p**, miR-381-3p, miR-654-5p, miR-2355-5p
IL-8 Signaling	7.39 x 10^–3^	11/183 (6.0%)	BCL2L1, CCND1, **CDH1**, GNG2, GNG4, HBEGF, IRAK2, **ITGAV**, **PLCB2**, RHOB, RHOG	**let-7i-5p**, **miR-29c-3p**, miR-146b-5p, miR-299-3p, **miR-342-5p**, miR-381-3p, **miR-503-5p**, miR-625-5p, miR-2355-5p
Clathrin-mediatedEndocytosis Signaling	7.68 x 10^–3^	11/184 (6.0%)	**ARRB1**, F2, **FGF2**, FGF5, GAK, HIP1, **MDM2**, PDGFB, SH3GL1, SH3GLB2, **TFRC**	**let-7i-5p**, **miR-29c-3p**, miR-133a, miR-148a-3p, **miR-342-5p**, miR-409-5p, miR-485-5p, miR-625-5p, miR-654-5p,
Wnt/β-catenin Signaling	1.02 x 10^–2^	10/166 (6.0%)	**AXIN2**, CCND1, **CDH1**, KREMEN1, LEF1, **MDM2**, **TCF4**, TGFB3, **TGFBR3**, WNT7B	**let-7i-5p**, **miR-29c-3p**, miR-128, miR-129-5p, **miR-146b-3p**, miR-148a-3p, miR-196b-5p, miR-205-5p, miR-299-3p, miR-376c-3p, **miR-342-5p**, miR-409-5p, miR-485-5p, **miR-503-5p**, miR-625-5p

The 480 differentially expressed genes targeted by miRNAs between SLKK and SLK cells were used as input for the Ingenuity pathway analysis (IPA). Here, we highlighted 4 of the top 10 enriched pathways that are relevant to KSHV pathogenesis. Columns identify the pathway name, its associated P-value, the ratio between dysregulated genes and genes in the pathway, which dysregulated target gene and miRNA are involved. Genes or miRNAs in bold and normal font were found up-regulated and down-regulated, respectively, by chronic KSHV infection.

## Discussion

In this study, we used next-generation sequencing to survey both mRNA and miRNA expression profiles in SLKK cells latently infected with KSHV as compared with the uninfected SLK cells. This allowed us to determine the degree to which changes in gene expression profiles could be attributed to changes in miRNA expression profiles. Looking at the broad trends in differential miRNA profiles, the majority of miRNAs that met our selection criteria (≥2 linear FC and P≤0.05) were down-regulated in the KSHV-infected cells. This is consistent with previous reports that lymphatic endothelial cells infected with KSHV and primary B-cells infected with Epstein-Barr virus (EBV) show a substantially higher number of down-regulated than up-regulated miRNAs [47,48]. One possible explanation was that KSHV-encoded miRNAs were produced at such a high level that they competitively interfered with the processing of the cellular miRNAs. However, in the SLKK cells, viral miRNAs made up only ~9% of the total miRNA fraction, suggesting that this was not the principal mechanism. Considering alternative explanations, other viruses have been shown to globally reduce cellular miRNA levels by the saturation of exportin-5 (XPO5), which is involved in transit of pre-miRNA to the cytoplasm [49], and it is possible that this may be occurring in KSHV-infected cells.

An unexpected finding was that more than a third of the significantly down-regulated miRNAs were located within 50kb of each other at the 14q32 miRNA cluster, which has previously been linked to cancer development. In fact, some of the 14q32 miRNAs that were easily detectable in the uninfected cells were undetectable in the KSHV-infected cells. We further validated the down-regulation of 14q32 miRNAs miR-409-3p, miR-409-5p, and miR-410 by qRT-PCR. A substantial down-regulation of 14q32 miRNAs has similarly been observed in PEL cell lines including BCBL-1, BC-1 and BC-3 [11]. Because of the extent of the down-regulation in the current study, we wondered whether a deletion of this region at the DNA level might have occurred, as it has been reported in a number of PEL cell lines and primary cases of PEL [33]. In SLKK cells however, DNA was present in each of eight regions that we probed, suggesting that repression of 14q32 miRNAs is largely due to epigenetic silencing at the DNA level or possibly post-transcriptional mechanisms. Also, the observation that 14q32 miRNAs are frequently down-regulated in the setting of KSHV infection and that it can occur either through chromosomal deletions or through other mechanisms suggests that down-regulation of these miRNAs are advantageous for survival of KSHV-infected cells.

A strength of the study is that we have simultaneously used deep sequencing to survey paired changes in miRNA and mRNA targets, in both infected and infected cell lines without transfection or other manipulation. However, a weakness of this approach is that miRNA changes are not the only factors that can modulate mRNA in this system and even where paired changes are seen, this does not prove causality; KSHV-encoded proteins, as well as manifestations of the host response, can also affect cellular mRNA through mechanisms other than the miRNA silencing machinery. Nonetheless, our analysis provides evidence to suggest that a substantial proportion of the observed mRNA changes were caused by miRNA dysregulation. We compiled an extensive list of strongly correlated miRNA-mRNA pairs. Of 48 miRNAs whose level met our cut-off criteria for modulation, there were predicted or experimentally determined targets that were also modulated in the SLKK cells, for a total of 1,117 targets. Of these, 73% (814 targets) were modulated in the direction predicted by the miRNA change. These modulated mRNAs include 36 mRNAs that had been previously reported as known targets for 9 miRNAs dysregulated in SLKK vs. SLK cells. We further transfected 3 miRNAs that were decreased in SLK or SLKK cells and examined changes in 5 respective mRNA targets, including 2 novel mRNAs (LIF for miR-708-5p and MDM2 for miR-409-5p). In each case, transfecting the miRNA resulted in a predicted decrease in the target gene(s). Moreover, based on our current understanding, changes in some of these mRNAs would benefit KSHV infection. For example, a decrease in caspase-2, a proapoptotic miR-708-5p target, would benefit maintenance of KSHV latent infection [44]. Taken together, these results are thus consistent with the hypothesis that changes in miRNAs are driving at least some of these mRNA changes. Furthermore, these results provide some additional confidence in the use of paired miRNA-Seq and mRNA-Seq in the SLK/SLKK system to identify mRNA targets that may be relevant to KSHV pathogenesis. However, it is unlikely that all of the changes in any given mRNA target are directly and uniquely caused by a specific miRNA, as biological systems are often redundant.

It is worth keeping in mind that while miRNA dysregulation could result from KSHV infection itself, it may also be due to a host response to infection, or external factors such as genetic drift in the established infected cell lines. Interestingly there was relatively little overlap between the miRNA changes observed here in latently-infected cells and those reported to occur in the first few hours after *de novo* KSHV infection, which would be expected to predominantly reflect the antiviral innate immune response or KSHV genes expressed early in infection [16]. This suggests that our findings are largely related to the effects of long-term KSHV latency. Looking at the miRNAs overexpressed here in SLKK cells, previous reports provide evidence linking certain miRNA changes to a direct effect of latent KSHV infection. For example, miR-146b has been shown to be induced by STAT3 [50], which is phosphorylated in cells with latent KSHV infection [51]. miR-146b in turn can down-regulate Toll-like receptors and other aspects of the immune response to pathogens and it may thus help KSHV evade immunity [52]. Moreover, a STAT3 knock-down study showed that STAT3 induces miR-21-3p and miR-29c-5p, which were found up-regulated in SLKK cells; and STAT3 represses miR-2276, which was found down-regulated in our study [53]. Also, while the change in miR-210 was not sufficient to meet our original P-value miRNA-Seq threshold, it was nonetheless found significantly up-regulated based on the Taqman assay. KSHV LANA has been shown to up-regulate hypoxia-inducible factor (HIF) [54], a direct inducer of miR-210 [55,56], so the increase in miR-210 was predicted based on this mechanism. How various cellular miRNAs are controlled by KSHV and the potential role that KSHV proteins may have at the post-transcriptional level are important questions that remain to be explored in the future.

Looking further at individual miRNAs dysregulated in SLKK cells and their target mRNAs, several stand out as potentially important in KSHV infection. For example, compared to SLK cells, SLKK cells manifested an increase in miR-708-5p along with a significant down-regulation of pro-apoptotic protease caspase-2 (CASP2). Through its induction of apoptosis, CASP2 can have an important anti-viral effect, and inhibition of this activity can thus promote KSHV infection. Also, miRNAs miR-485-5p and miR-409-5p are down-regulated in KSHV-infected cells and this directly correlates with the up-regulation of their predicted target, MDM2, which mediates the degradation of p53 [57,58], an inducer of apoptosis. By this mechanism, MDM2 may suppress virus-induced apoptosis, thereby increasing the survival of KSHV-infected cells. Two other down-regulated miRNAs, miR-299-3p and miR-381-3p, are predicted to suppress expression of integrin V (ITGAV) (Table 2 and Fig 6), which was in turn up-regulated in SLKK cells. ITGAV has been shown to enhance the ability of KSHV-infected cells to infect new cells through binding to the extracellular matrix [59]. Interestingly, miR-409-5p, miR-299-3p, and miR-381-3p are all located in the 14q32 cluster that is broadly down-regulated in SLKK cells as well as PEL cells [33]. Additional studies will be needed to clarify the biological effects of these various miRNA changes.

In this study, we also used pathway analysis tools to identify some of the most affected pathways. Overall, we found 45 miRNAs and 480 paired mRNA targets that were either experimentally determined or highly predicted and whose changes inversely correlated with the mRNA changes. Of these, 17 miRNAs and 28 putative mRNA targets have been previously identified as being involved in pathogen-related signaling (Fig 6). We also noted several fundamental pathways that were significantly enriched in the 480 mRNA targets. These include the regulation of the epithelial-to-mesenchymal transition (EMT) pathway, IL-8 signaling, clathrin-mediated endocytosis signaling and Wnt/-catenin signaling (Table 4).

We used SLK and SLKK cells in the current studies because they provided the ability to compare KSHV-infected and uninfected cells and because of the tight control of KSHV latency in these cells. Experimentally derived from a KS biopsy, the uninfected SLK cell line was initially thought to have endothelial features matching those of KS and it was anticipated to become a KS cell model since it can be stably and latently infected by KSHV [21,22]. However, it was later shown to be indistinguishable from Caki-1, a clear-cell renal-cell carcinoma line of epithelial origin [20]. It is worth noting that SLK cells stably infected with recombinant rKSHV.219 (SLKK) were selected over time to only consist of KSHV-infected cells and some of the changes in SLKK cells as compared to SLK cells could be the result of genetic drift. However, some of the miRNA changes observed in the SLKK cell line were also observed upon *de novo* infection and therefore genetic drift could not account for all the changes. Thus, the SLK/SLKK system remains a valuable model to study host effects from latent KSHV infection and a number of changes observed here have also been observed in other studies of miRNA or mRNA expression in KSHV-infected cells [18]. This said, a number of complex factors may be contributing to specific observed changes, and additional studies will be needed to tease out these relationships.

In summary, we used deep sequencing to examine the small RNAs and mRNAs expressed by SLKK and SLK cells, and we identified a number of miRNA-target mRNA pairs that play a significant role in KSHV pathogenesis and in some cases, KSHV-induced diseases. We also observed that the majority of cellular miRNAs that are significantly altered by KSHV are down-regulated, including most members of the 14q32 miRNA cluster, a genomic locus linked to cancer. To our knowledge, this is the first comparative study correlating changes at the miRNA and mRNA level between cells with latent KSHV infection and uninfected cells. This survey can help inform additional studies to better understand the mechanisms responsible for the specific changes observed and their role in the establishment of KSHV infection, the host response to this infection, and the pathogenesis of KSHV-induced tumors.

## Supporting Information

S1 Fig Expression of miRNAs in the analyzed small RNA libraries. A. Scatterplot of the transcriptome of KSHV-infected versus uninfected cells. The X-axis represents the average of the normalized miR read counts across all samples. The Y-axis represents the log-transformed expression fold change between SLKK and SLK cells. Blue dots are differentially expressed genes with a *P* <0.05, while grey dots are those with a *P* ≥0.05. B. Heatmap of miRNA changes between SLK and SLKK cells in the six individual replicates (three SLK and three SLKK). Using uncentered Pearson correlation as the distance metric, we generated an unsupervised hierarchical heatmap where each row represents a miRNA and each column represents a biological replicate. Red, black and green denote low, median and high relative miRNA expression, respectively.
(TIFF)

S2 Fig Genomic map of the cluster of miRNAs at the 14q32 locus. The miRNA cluster B of the 14q32 imprinted region is illustrated here. 34 out of 41 14q32 miRNAs (83%) have at least one arm that is down-regulated. Also, counting the 3p and 5p arms separately, 42 out of 111 (38%) miRNAs that are down-regulated by KSHV infection are located within this miRNA cluster. Below the line, in black are miRNAs that are significantly down-regulated by at least -1 log_2_-transformed FC (*P* <0.05). miRNAs above the line in grey are not significantly altered by KSHV infection. Those above the line in black are not expressed in the SLK/SLKK cell model.
(TIFF)

S3 Fig DNA presence across the 14q32 cluster. We interrogated several regions of the genomic DNA between KSHV-infected uninfected samples. This qualitative assessment shows that all regions were present in both samples.
(TIFF)

S4 Fig Expression of mRNAs in the poly-A enriched RNA libraries. A. Scatterplot of the transcriptome of KSHV-infected versus uninfected cells. The X-axis represents the average of the normalized read counts across all samples. The Y-axis represents the log_2_-transformed expression fold change between SLKK and SLK cells. Blue dots are differentially expressed genes with a FDR <0.05, while grey dots are those with a FDR ≥0.05. B. Heatmap of mRNA changes between SLK and SLKK cells in the six individual replicates (three SLK and three SLKK). Using uncentered Pearson correlation as the distance metric, we generated an unsupervised hierarchical heatmap where each row represents a mRNA and each column represents a biological replicate. Red, black and green denote low, median and high relative miRNA expression, respectively.
(TIFF)

S1 Table Primer sequences for PCR amplification of the 14q32 genomic region. Eight individual regions (~450bp) of the genomic DNA at 14q32 were probed using these custom-designed primers. Columns identify the primer name, direction, sequence as well as amplicon size.
(DOCX)

S2 Table KSHV miRNA distribution in SLKK cell line. This table shows the read count and percentage representation of 25 mature KSHV miRNAs expressed in SLKK cells.
(DOCX)

S3 Table Reads statistics for miRNAs expressed in KSHV-positive SLKK cells. Three independent experiments are displayed (A, B and C). Columns identify the replicate number, raw read counts for human or mature miRNAs, number of mapped miRNAs per species, which percentage these KSHV reads represented for each replicate or overall. These counts were obtained by mapping reads to either KSHV mature miRNAs or human mature miRNAs.
(DOCX)
