# Supplementary figures and images for: Next-Generation Sequencing Analysis Reveals Differential Expression Profiles of MiRNA-mRNA Target Pairs in KSHV-Infected Cells
Source: PLoS One. 2015 May 5;10(5):e0126439. doi: 10.1371/journal.pone.0126439 (PMC4420468; doi:10.1371/journal.pone.0126439)

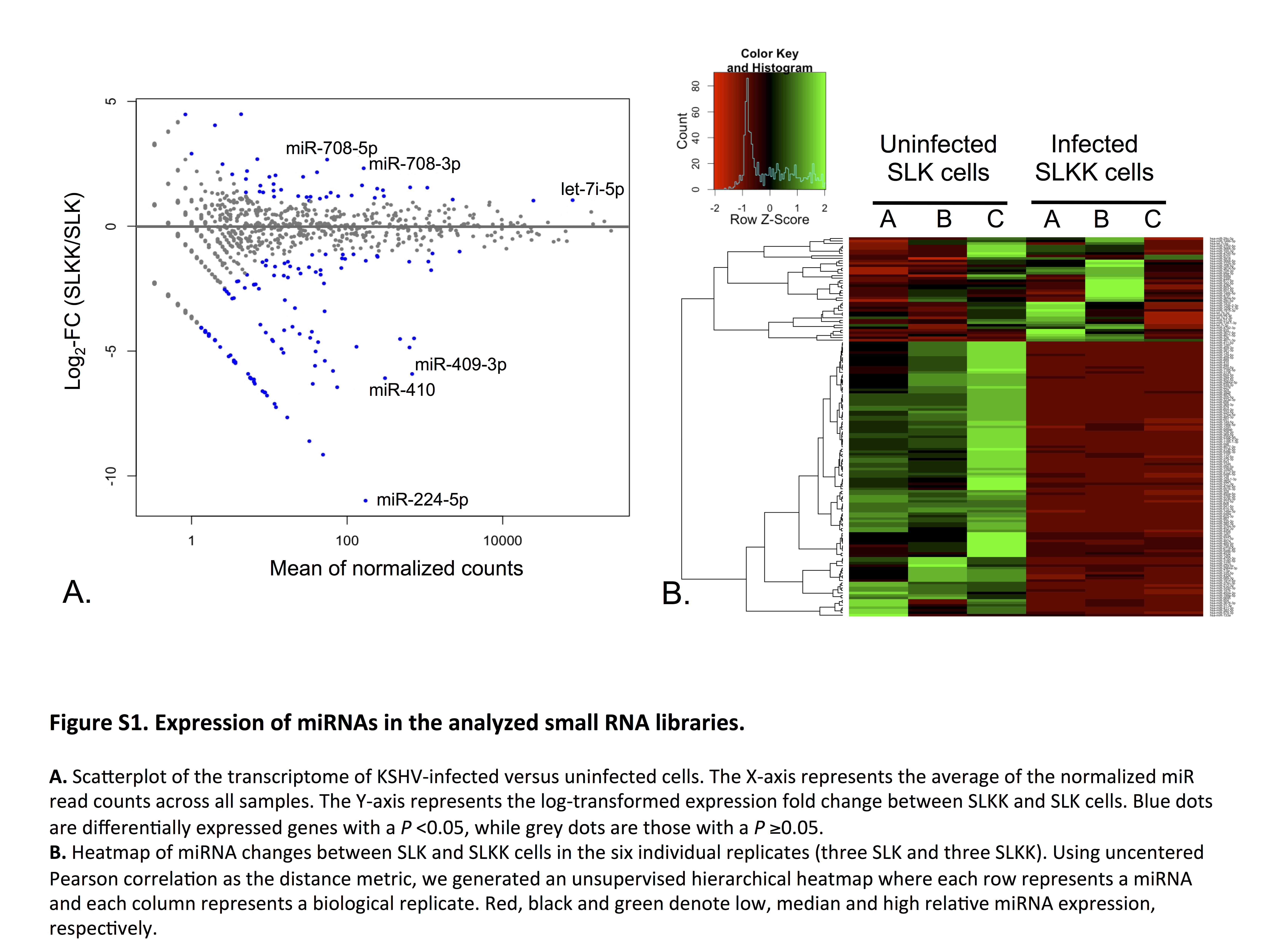

Supplement: S1 Fig — A. Scatterplot of the transcriptome of KSHV-infected versus uninfected cells. The X-axis represents the average of the normalized miR read counts across all samples. The Y-axis represents the log-transformed expression fold change between SLKK and SLK cells. Blue dots are differentially expressed genes with a P <0.05, while grey dots are those with a P ≥0.05. B. Heatmap of miRNA changes between SLK and SLKK cells in the six individual replicates (three SLK and three SLKK). Using uncentered Pearson correlation as the distance metric, we generated an unsupervised hierarchical heatmap where each row represents a miRNA and each column represents a biological replicate. Red, black and green denote low, median and high relative miRNA expression, respectively. (TIFF) [file pone.0126439.s001.tiff]

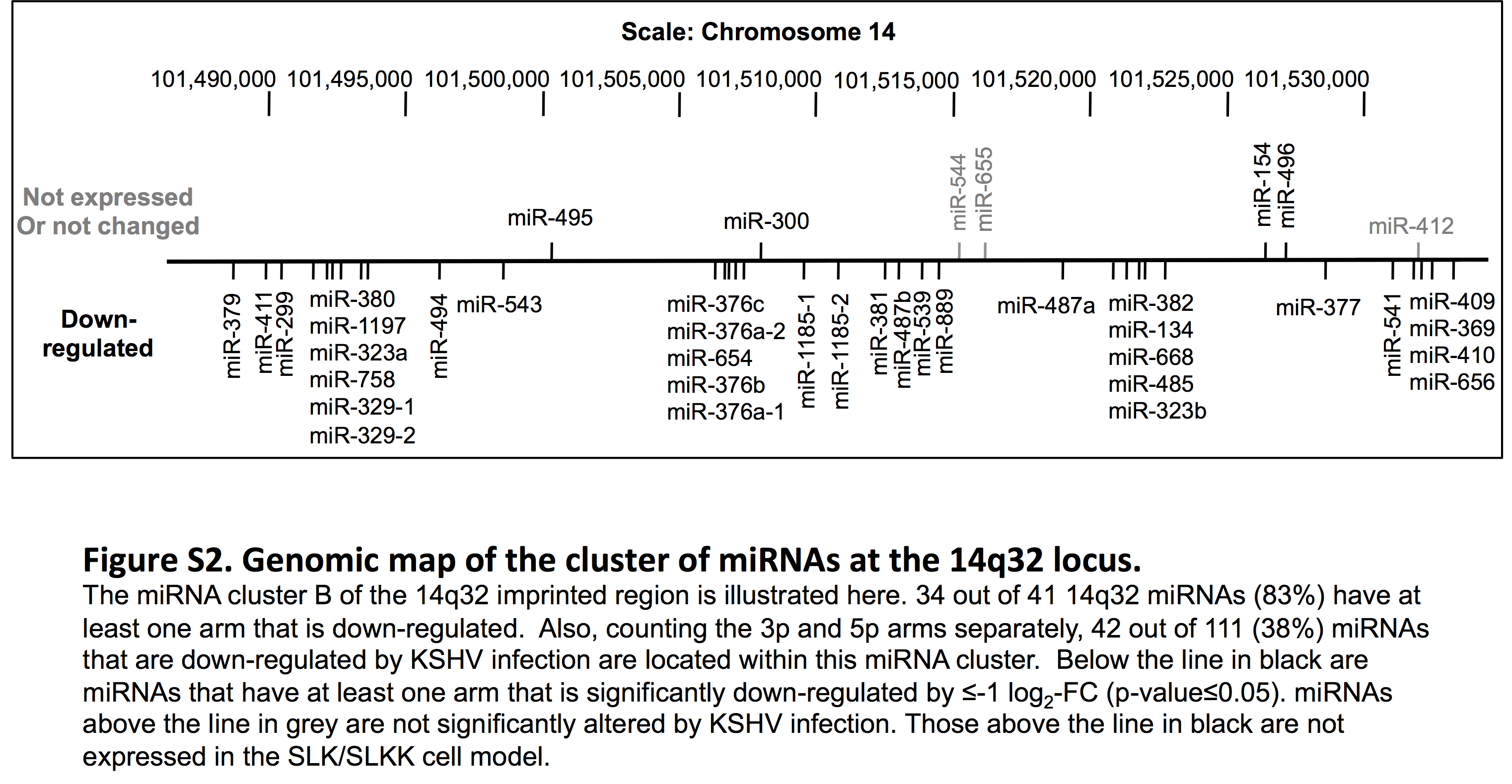

Supplement: S2 Fig — The miRNA cluster B of the 14q32 imprinted region is illustrated here. 34 out of 41 14q32 miRNAs (83%) have at least one arm that is down-regulated. Also, counting the 3p and 5p arms separately, 42 out of 111 (38%) miRNAs that are down-regulated by KSHV infection are located within this miRNA cluster. Below the line, in black are miRNAs that are significantly down-regulated by at least -1 log2-transformed FC (P <0.05). miRNAs above the line in grey are not significantly altered by KSHV infection. Those above the line in black are not expressed in the SLK/SLKK cell model. (TIFF) [file pone.0126439.s002.tiff]

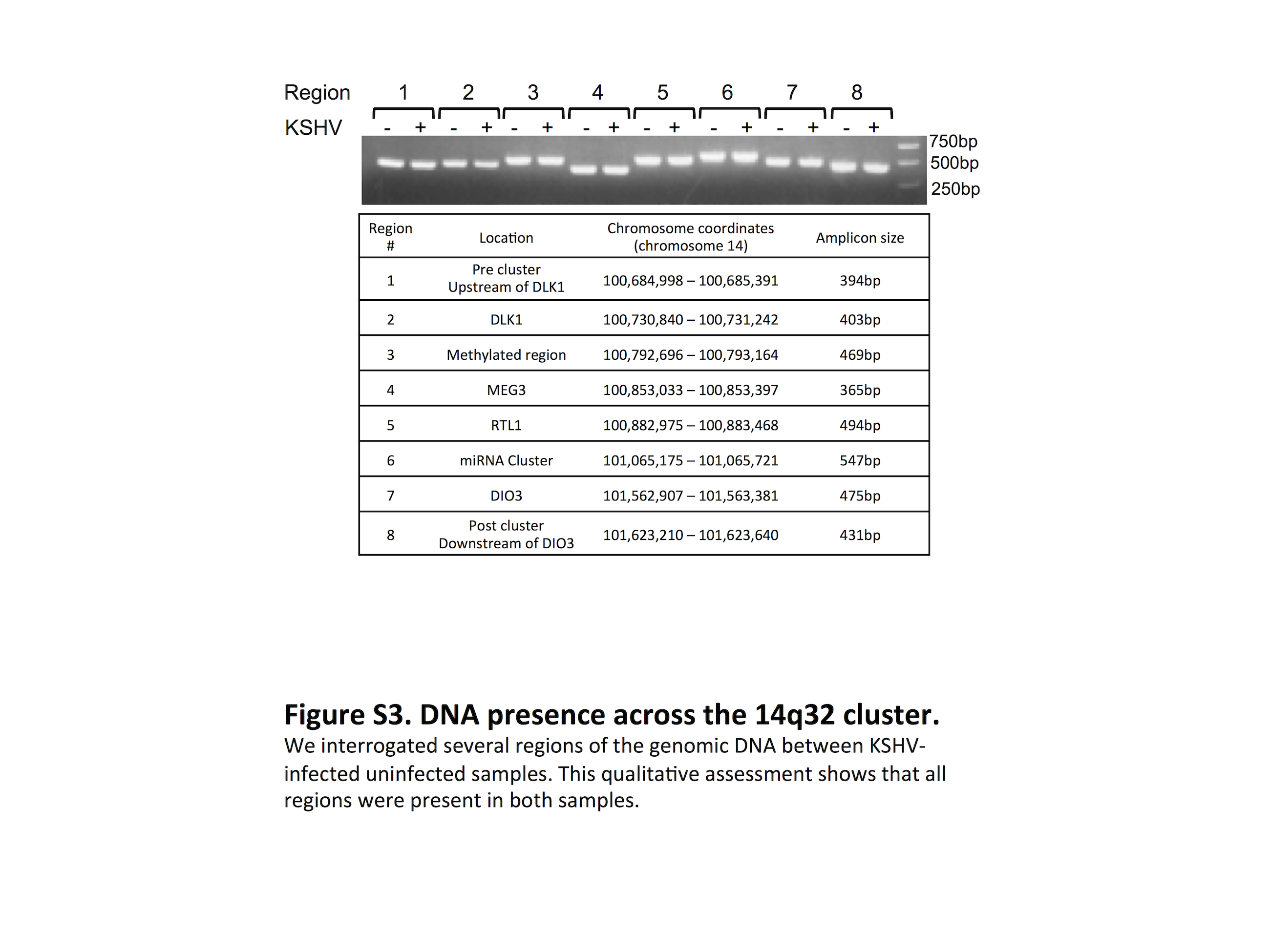

Supplement: S3 Fig — We interrogated several regions of the genomic DNA between KSHV-infected uninfected samples. This qualitative assessment shows that all regions were present in both samples. (TIFF) [file pone.0126439.s003.tiff]

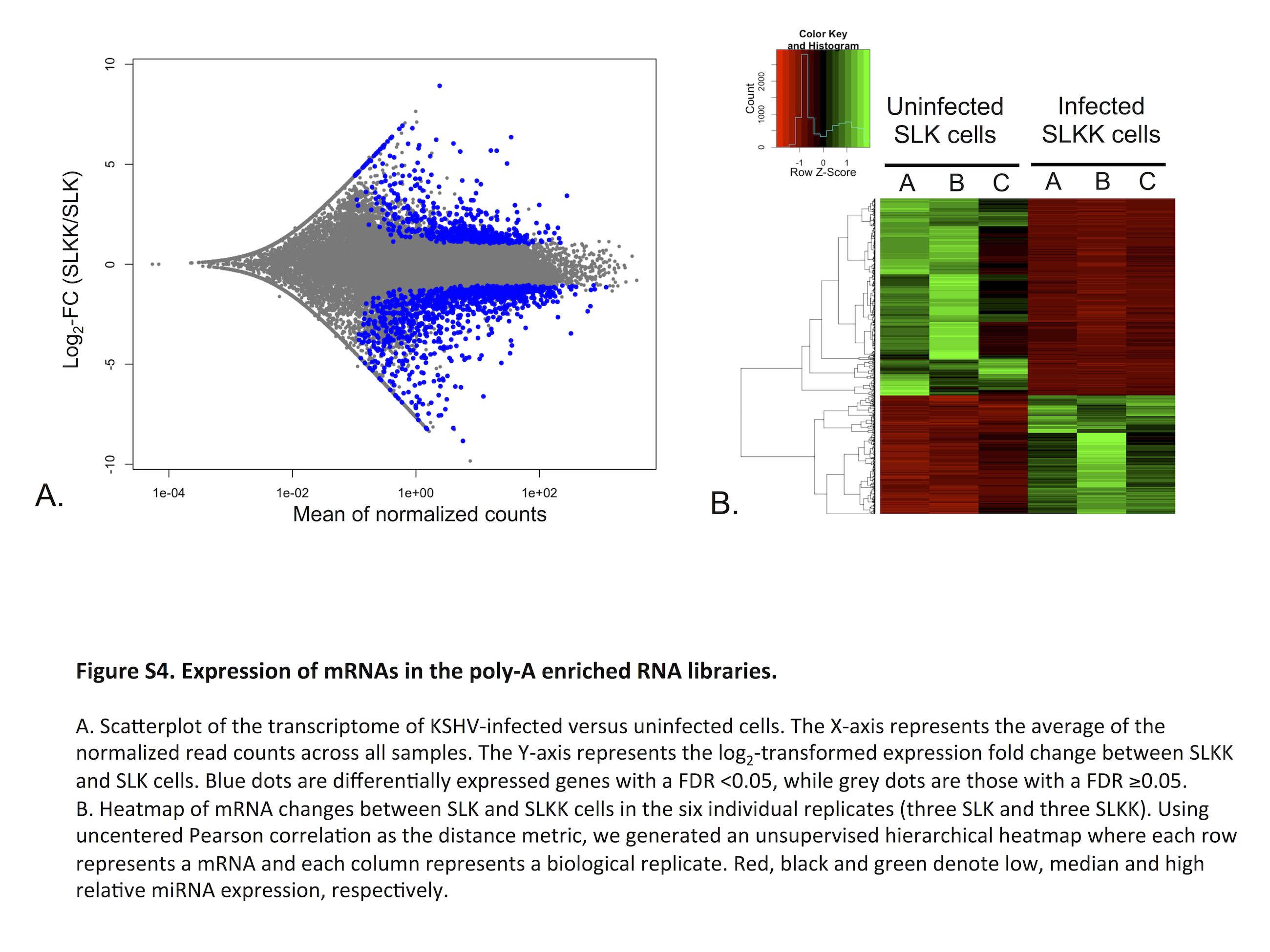

Supplement: S4 Fig — A. Scatterplot of the transcriptome of KSHV-infected versus uninfected cells. The X-axis represents the average of the normalized read counts across all samples. The Y-axis represents the log2-transformed expression fold change between SLKK and SLK cells. Blue dots are differentially expressed genes with a FDR <0.05, while grey dots are those with a FDR ≥0.05. B. Heatmap of mRNA changes between SLK and SLKK cells in the six individual replicates (three SLK and three SLKK). Using uncentered Pearson correlation as the distance metric, we generated an unsupervised hierarchical heatmap where each row represents a mRNA and each column represents a biological replicate. Red, black and green denote low, median and high relative miRNA expression, respectively. (TIFF) [file pone.0126439.s004.tiff]
