# Supplementary material for: Next-Generation Sequencing Analysis Reveals Differential Expression Profiles of MiRNA-mRNA Target Pairs in KSHV-Infected Cells
Source: PLoS One. 2015 May 5;10(5):e0126439. doi: 10.1371/journal.pone.0126439 (PMC4420468; doi:10.1371/journal.pone.0126439)
Supplement: S1 Table — Eight individual regions (~450bp) of the genomic DNA at 14q32 were probed using these custom-designed primers. Columns identify the primer name, direction, sequence as well as amplicon size. (DOCX) [file pone.0126439.s005.docx]

**S1 Table.** **Primer sequences for PCR amplification of the 14q32 genomic region.**

| Region | Primer name | Direction | Sequence | Amplicon size |
| --- | --- | --- | --- | --- |
| 1 | Upstream DLK1 | F | TGGGAGCTCAAGGTCAGTCT | 394bp |
|  |  | R | AAAAGTAGGGAAGCACCCGT |  |
| 2 | DLK1 | F | CCATCAACACAACGTTCAGG | 403bp |
|  |  | R | TCTCTGATGATGGAGACCCC |  |
| 3 | Methylation Region | F | ACCACCTTAGATGCCGTGTC | 469bp |
|  |  | R | TGCTCAGCTCTGTCTCCTGA |  |
| 4 | MEG3 | F | GGATGCTGAGATTCGGGATA | 365bp |
|  |  | R | TAGGAACACAACGGGACACA |  |
| 5 | RTL1 | F | AAGTTCGCCCAAGAGCACTA | 494bp |
|  |  | R | TCGCTGTGATCACTGTCTCC |  |
| 6 | miRNA Cluster | F | TCCTTGGAGAGTGTGAGGCT | 547bp |
|  |  | R | CCTACCCTCAAAAGGGCTTC |  |
| 7 | DIO3 | F | TGTTTCCTGTCCCTGGTAGG | 475bp |
|  |  | R | ACACTCACCAAATGGCCTTC |  |
| 8 | Downstream DIO3 | F | AGGGGAACAACAGTGTGGAG | 431bp |
|  |  | R | TGGAGCTGGGAGAAGACACT |  |

Eight individual regions (~450bp) of the genomic DNA at 14q32 were probed using these custom-designed primers. Columns identify the primer name, direction, sequence as well as amplicon size.
