# Supplementary material for: Next-Generation Sequencing Analysis Reveals Differential Expression Profiles of MiRNA-mRNA Target Pairs in KSHV-Infected Cells
Source: PLoS One. 2015 May 5;10(5):e0126439. doi: 10.1371/journal.pone.0126439 (PMC4420468; doi:10.1371/journal.pone.0126439)
Supplement: S2 Table — This table shows the read count and percentage representation of 25 mature KSHV miRNAs expressed in SLKK cells. (DOCX) [file pone.0126439.s006.docx]

**S2 Table.** **KSHV miRNA distribution in SLKK cell line.**

| **KSHV miRNA** | **Count** | **Percentage of KSHV** |
| --- | --- | --- |
| KSHV-miR-K12-10a-3p | 405,293 | 40.53% |
| KSHV-miR-K12-4-3p | 261,969 | 26.20% |
| KSHV-miR-K12-8-3p | 126,885 | 12.69% |
| KSHV-miR-K12-3-5p | 51,448 | 5.14% |
| KSHV-miR-K12-10b | 46,503 | 4.65% |
| KSHV-miR-K12-7-3p | 18,871 | 1.89% |
| KSHV-miR-K12-2-5p | 17,029 | 1.70% |
| KSHV-miR-K12-6-3p | 154,08 | 1.54% |
| KSHV-miR-K12-11-3p | 14,417 | 1.44% |
| KSHV-miR-K12-12-5p | 11,199 | 1.12% |
| KSHV-miR-K12-4-5p | 10,389 | 1.04% |
| KSHV-miR-K12-6-5p | 5,124 | 0.51% |
| KSHV-miR-K12-3-3p | 3251 | 0.33% |
| KSHV-miR-K12-12-3p | 2,938 | 0.29% |
| KSHV-miR-K12-1-5p | 2,789 | 0.28% |
| KSHV-miR-K12-5-3p | 2,657 | 0.27% |
| KSHV-miR-K12-9-3p | 1,568 | 0.16% |
| KSHV-miR-K12-8-5p | 962 | 0.10% |
| KSHV-miR-K12-9-5p | 562 | 0.06% |
| KSHV-miR-K12-7-5p | 401 | 0.04% |
| KSHV-miR-K12-2-3p | 182 | 0.02% |
| KSHV-miR-K12-10a-5p | 139 | 0.01% |
| KSHV-miR-K12-1-3p | 7 | 0.00% |
| KSHV-miR-K12-5-5p | 3 | 0.00% |
| KSHV-miR-K12-11-5p | 5 | 0.00% |

This table shows the read count and percentage representation of 25 mature KSHV miRNAs expressed in SLKK cells.
