# Supplementary material for: Next-Generation Sequencing Analysis Reveals Differential Expression Profiles of MiRNA-mRNA Target Pairs in KSHV-Infected Cells
Source: PLoS One. 2015 May 5;10(5):e0126439. doi: 10.1371/journal.pone.0126439 (PMC4420468; doi:10.1371/journal.pone.0126439)
Supplement: S3 Table — Three independent experiments are displayed (A, B and C). Columns identify the replicate number, raw read counts for human or mature miRNAs, number of mapped miRNAs per species, which percentage these KSHV reads represented for each replicate or overall. These counts were obtained by mapping reads to either KSHV mature miRNAs or human mature miRNAs. (DOCX) [file pone.0126439.s007.docx]

**S3 Table.** **Reads statistics for miRNAs expressed in KSHV-positive SLKK cells.**

| **Replicate** | **Human**  **miR count** | **Matches per human miR** | **KSHV**  **miR count** | **Matches per KSHV miR** | **Total**  **miR count** | **Percentage of Total** |
| --- | --- | --- | --- | --- | --- | --- |
| SLKK A | 956,091 | 468 | 147,841 | 5,914 | 1,103,932 | 13.4% |
| SLKK B | 1,561,579 | 765 | 126,564 | 5,063 | 1,688,143 | 7.5% |
| SLKK C | 642,955 | 315 | 50,288 | 2,012 | 693,243 | 7.3% |
| **Average** | **1,053,542** | **516** | **108,231** | **4,329** | **1,161,773** | **9.4%** |

Three independent experiments are displayed (A, B and C). Columns identify the replicate number, raw read counts for human or mature miRNAs, number of mapped miRNAs per species, which percentage these KSHV reads represented for each replicate or overall. These counts were obtained by mapping reads to either KSHV mature miRNAs or human mature miRNAs.
